# Prevalence and assessment of self-disorders in the schizophrenia spectrum: a systematic review and meta-analysis

**DOI:** 10.1038/s41598-022-05232-9

**Published:** 2022-01-21

**Authors:** Sam Burgin, Renate Reniers, Clara Humpston

**Affiliations:** 1 University of Birmingham Medical School, University of Birmingham, Birmingham, B15 2TT UK; 2 Institute of Clinical Sciences, University of Birmingham, Birmingham, B15 2TT UK; 3 Institute for Mental Health, University of Birmingham, Birmingham, B15 2TT UK

**Keywords:** Psychology, Human behaviour

## Abstract

Self-disorders have been proposed as the “clinical core” of the schizophrenia spectrum. This has been explored in recent studies using self-disorder assessment tools. However, there are few systematic discussions of their quality and utility. Therefore, a literature search was performed on Medline, Embase, PsychINFO, PubMed and the Web of Science. Studies using these assessment tools to explore self-disorders within schizophrenia spectrum disorders (SSDs) were included. A meta-analysis was performed on the outcomes of total self-disorder score and odds ratios of self-disorders, using Comprehensive Meta-Analysis software. Weighted pooled effect sizes in Hedge’s *g* were calculated using a random-effects model. 15 studies were included, giving a sample of 810 participants on the schizophrenia spectrum. Self-disorders showed a greater aggregation within schizophrenia spectrum groups compared to non-schizophrenia spectrum groups, as measured with the Bonn Scale for the Assessment of Basic Symptoms (Hedge’s *g* = 0.774, *p* < 0.01) and Examination of Anomalous Self-Experiences (Hedge’s *g* = 1.604, *p* < 0.01). Also, self-disorders had a greater likelihood of occurring within SSDs (odds ratio = 5.435, *p* < 0.01). These findings help to validate self-disorders as a core clinical feature of the broad schizophrenia spectrum.

## Introduction

The schizophrenia spectrum describes a range of psychotic disorders characterised by continuous or episodic positive and negative symptoms, as well as cognitive impairment^1,2^. Although checklists such as the DSM-5 aid diagnosis and categorisation of schizophrenia spectrum disorders (SSD), they fail to give healthcare professionals a nuanced understanding of the ‘clinical core’ of these disorders^3^.

The concept of self-disorders (SD) was derived from a subgroup of basic symptoms (BS), and BS may be seen as providing historical or conceptual influence on the later SD^4^. BSs are subtle, subclinical, and subjectively experienced disturbances of mental processes^5–7^. They include thought interference, thought block and a disruption to abstract thinking^5^. It may be somewhat intuitive to consider BSs as precursors to first-rank symptoms such as thought insertion, although from a historical-conceptual perspective, first-rank symptoms were developed prior to the BS^5,6^. A detailed description of BSs can be found in Schultze-Lutter et al.^7^. Though the aetiology is poorly understood, the Early Heidelberg School’s perceptual anomalies model for self-disturbances (the preferred term over ‘self-disorders’) proposes these abnormalities arise in sensory processing at an unconscious level^5,8^. The Early Heidelberg School of Psychiatry first developed and systematically described “self-disturbances” (*Ichstörungen* in German) in schizophrenia. Kurt Schneider later incorporated many self-disturbances in his first-rank symptoms. This approach has been further developed by several pioneering authors and is actively used today^8–11^. By applying this concept alongside in-depth interviews of patients with schizophrenia, the Bonn Scale for the Assessment of Basic Symptoms (BSABS)^12^, and later Schizophrenia Proneness Instrument (SPI), were developed^13^. A subgroup of BSABS items explore BSs relating to anomalies in one’s subjective self-experience.

The ipseity disturbance model developed the concept of SDs, or anomalous self-experiences (ASEs)^7^. The ipseity disturbance model defines SDs as psychiatric phenomena characterised by a trait-like, persistent disruption in the tacit, pre-reflective level of selfhood, known as the minimal self^14^. The minimal self, sometimes used interchangeably with ipseity, is the basic level of selfhood where a subject’s emotions, experiences and actions are given first-person ownership, agency, and awareness^15^. This distinguishes SDs in the schizophrenia spectrum from self-disorder-like phenomena seen in other conditions such as personality disorders^16–18^. Proponents of the ipseity disturbance model combined qualitative findings from patients with SSDs with a subgroup of BSABS items to create the Examination of Anomalous Self-experience (EASE)^7,19^.

Studies utilising the BSABS and EASE have shown the aggregation of certain SDs within SSDs^20–22^. A recent study by Koren et al.^23^ provides longitudinal evidence to corroborate this cross-sectional association between SDs and SSDs^23^. In this study, a greater total SD score was associated with a greater risk of conversion to non-affective psychosis (NAP) and schizotypal disorder, when compared with other psychiatric disorders. Other studies have demonstrated statistically insignificant differences in SD scores between schizophrenia and schizotypal disorders, validating the theory that SDs are a core feature of the broad schizophrenia spectrum and represent a clinical vulnerability phenotype^24,25^.

Systematic reviews have begun to emerge within this field, notably a recent meta-analysis by Raballo et al.^26^ and a systematic review by Henriksen, Raballo, and Nordgaard^27^. This meta-analysis reported the selective aggregation of SDs within SSDs, when compared to other mental illnesses (OMI) and healthy controls (HC)^26^. This meta-analysis also reported evidence suggesting that SDs may be a marker of vulnerability for conversion to full-blown psychosis within the schizophrenia spectrum. Like the present study, Raballo et al.’s meta-analysis explored the extent to which SDs express a specificity for SSDs. However, unlike Raballo et al.’s study, this systematic review also poses questions of how likely SDs are to occur within the schizophrenia population and how clinically useful current SD assessment tools are. These questions highlight gaps in current literature, including recent meta-analyses. Raballo et al.^26^ report a methodology in line with the PRISMA guidelines and utilise data such as mean differences in SD score, mirroring this study. However, both studies vary in eligibility criteria, with^26^ including studies with clinically high-risk (CHR) and child/adolescent groups. Although follow-up studies have demonstrated that SDs are temporally stable traits in help-seeking adolescents which also predict later transition to a diagnosis of SSDs^23^, these groups have been excluded in the current analysis given the potential of added heterogeneity and uncertainties in diagnoses, at least cross-sectionally, in adolescent samples. Thus, whilst the pooled effect sizes this meta-analysis generates may have reduced power, they should be more precise and enable the generation of more accurate conclusions. In contrast to Raballo et al.^26^’s meta-analysis^26^, this meta-analysis separately explores BSABS and EASE studies, which should facilitate the comparison of these assessment tools and improve precision.

This systematic review and meta-analysis propose that self-disturbances or self-disorders provide a promising avenue for gaining a better subjective understanding of the core phenomena of SSDs^3^. This study aims to answer the following questions. Firstly, what is the likelihood of presenting self-disorders or self-disturbances among the schizophrenia spectrum population? Secondly, what is the difference in self-disorder scores between schizophrenia spectrum groups and other non-schizophrenia spectrum groups? Thirdly, what is the clinical utility of current assessment tools in identifying self-disorders among schizophrenia spectrum groups?

## Results

### Study selection

**Figure 1:**
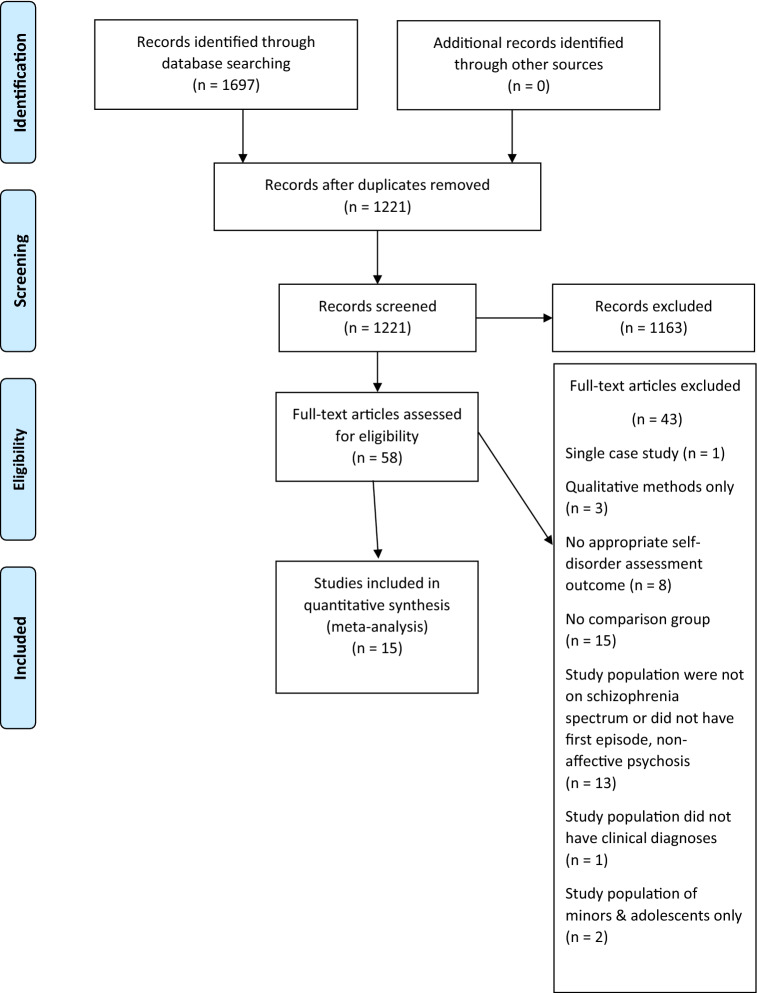
PRISMA flowchart for study selection.

Following application of the eligibility criteria, two open-label cohort/follow-up and 13 open-label cross-sectional observational studies were included in the systematic review and meta-analysis (PRISMA Flowchart, Fig. 1).

### Study characteristics

Table 1 shows the characteristics of all included studies. Roughly half of included studies were performed in inpatient (five studies) and outpatient units (seven studies). Three studies were set in a combined inpatient and outpatient unit. The geographical setting for included studies varied. However, Denmark was the setting of the largest proportion of studies (eight studies). The other studies were set in Norway (two studies), Melbourne (three studies), Portugal (one study), and Italy (one study). Of the included studies, six used the BSABS and nine used the EASE for assessment of SDs. Studies using the EASE varied in how the SD score outcome was measured. Six of the EASE studies reported dichotomous scores, two of the nine EASE studies reported continuous scores, and one reported both scores.

**Table 1:** Summary table of included studies reporting the following: main author(s), geographical setting, criterion/instrument for diagnosis, SD assessment tool, type of sample, sample size, mean total SD score, odds ratio of SD, descriptive psychopathology, risk of bias rating, quality of evidence rating, and key findings.

Author (s)	Year	Geographical setting	Criterion and instrument for diagnosis	Self-disorder assessment tool used	Type of sample	Sample size	Mean total SD score (s.d.)	Odds ratio of SD	Descriptive psychopathology	Risk of bias rating	Quality of evidence rating	Key findings
Handest and Parnas^28^	2005	Copenhagen, Denmark Inpatient unit	OPCRIT ICD-10 DSM-IV	BSABS	SZ + NAP SPD OMI	51 50 50 151	5.19 (3.94) 4.71 (3.94) 2.25 (2.51)		Psychosis group: Mean age = 25.3 years Mean duration of illness = 4.5 years Mean duration of psychosis = 2.3 years Diagnoses include SZ (41), acute psychoses, schizoaffective (1), delusional disorder (1) Schizotypal group: Mean age = 24.6 years Mean duration of illness = 7.0 years Mean duration of psychosis = 0 years OMI group: Mean age = 26.2 years Mean duration of illness = 7.5 years Mean duration of psychosis = 0 years Diagnoses include affective illness, OCD, anxiety, eating disorders, personality disorders No significant IQ, age differences or gender differences (except in SPD group) Psychosis group had lower GAF compared to others, longer duration of untreated illness, longer social and work dysfunction	Low	Moderate	Those with SPD scored intermediately between OMI and psychosis group on positive and negative symptoms, emotional contact disorders and formal thought disorder scales Participants in the SPD and schizophrenia group scored the same for ASEs ICD-10 SPD appears to be a milder, less psychotic variant of schizophrenia with no clear line demarcating the two
Haug et al.^29^	2012	Multi-center, Norway Inpatient unit	DSM-IV	EASE	SZ NAP BD	57 13 21 91 Dropped to 90	25.30 (9.60) 11.50 (8.70) 6.3 (4.8)		SZ group: Mean age = 25.4 (7.3) M/F ratio = 29/28 Duration of psychosis (median) = 122 (4–2040) Mean GAF symptom = 34.9 (8.9) Mean PANSS = 76.4 (16.8) Mean YMRS = 6.4 (4.6) Mean CDSS = 9.4 (5.9) BD group: Mean age = 24.4 (7.7) M/F ratio = 8/13 Duration of psychosis (median) = 2 (0–674) Mean GAF symptoms = 51.0 (14.6) Mean PANSS = 53.5 (15.9) Mean YMRS = 3.3 (5.1) OMI group: Mean age = 23.8 (6.9) M/F ratio = 11/2 Duration of psychosis (median) = 78 (8–312) Mean GAF symptoms = 38.0 (3.1) Mean PANSS = 70.2 (9.7) Mean YMRS = 4.6 (4.1)	Moderate	Moderate	The presence of self-disorders showed a strong correlation with SZ diagnosis (statistically significant) compared to BD or OMI. Even when corrected for symptomatic, demographic and functional differences The odds ratio for a diagnosis of SZ vs BD was 1.4 for each step increase of each EASE item/total score The odds ratio for a diagnosis of SZ vs OMI was 1.2 for each step increase of each EASE item/total score
Madeira et al.^30^	2019	Lisbon, Portugal Inpatient unit	DSM-IV	EASE EAWE	FEP HC	24 (21 ASE) 24 (11 ASE) 48	20.00 (17.14) 1.00 (1.74)		Whole group: Mean age = 27.29 (1.44) M/F ratio = 34/14 Primary school education = 25.5% Bach/Master degree education = 74.5% Ethnicity Caucasian = 89.6% Ethnicity Other = 10.4% FEP group: Mean age = 27.00 (9.807) M/F ratio = 22/2 Primary school education = 67.7% Bach/Master degree education = 33.3% Ethnicity Caucasian = 87.5% Ethnicity Other = 12.5% HC group: Mean age = 27.58 (10.325) M/F ratio = 17/7 Primary school education = 52.1% Bach/Master degree education = 47.9% Ethnicity Caucasian = 91.7% Ethnicity Other = 8.3%	Moderate	Moderate	Participants with non-affective FEP on average had higher total EASE and EAWE scores compared to HCs EASE and EAWE scores showed strong correlations even after removal of duplicate items *r* = 0.927 (95% CI 0.510–0.650, *p* = 0.000 15/24 FEP participants reported anomalous world experiences (AWEs) with an average score of 19.96 (21.44). 4/24 HC participants reported AWEs with an average score < 6 21/24 FEP participants reported ASEs with an average score of 20.00 (17.14). 11/24 HC participants reported ASEs with an average score < 6 Variable standard deviations show wide variability across participants
Nelson et al.^16^	2013	Melbourne, Australia Inpatient and Outpatient unit	DSM-IV	EASE	SZ OMI	8 8 16	22.50 (12.98) D 73.50 (46.22) C 10.25 (7.15) D 33.63 (17.48) C		Whole group: Mean age = 21.63 (3.7) M/F ratio = 10/6 Currently employed or studying Y/N = 7/9 Married/single = 0/16 History of psychiatric treatment Y/N = 15/1 Family history Y/N = 6/10 Mean duration of illness = 3.06 (1.77) SZ group: Mean age = 22.25 (4.23) M/F ratio = 4/4 Currently employed or studying Y/N = 1/7 Married/single = 0/8 History of psychiatric treatment Y/N = 7/1 Family history Y/N = 4/4 Mean duration of illness = 3.50 (2.33) SZ (5), schizophreniform (2), schizoaffective (1) OMI group: Mean age = 21.00 (3.30) M/F ratio = 6/2 Currently employed or studying Y/N = 6/2 Married/single = 0/8 History of psychiatric treatment Y/N = 8/0 Family history Y/N = 2/6 Mean duration of illness = 2.63 (0.92) Psychotic disorder NOS (5), mood disorder + psychosis (1), BD + psychosis (1), substance-induced psychotic disorder (1)	Moderate	Low	Participants with a schizophrenia spectrum diagnosis had significantly higher EASE total scores Scores were significantly different in the self-awareness and presence, bodily experiences and demarcation/transitivism domains
Nelson et al.^31^	2020	Melbourne, Australia Inpatient and Outpatient unit	DSM-IV CAARMS	EASE	FEP UHR HC	39 50 34 123	78.74 (29.82) 63.30 (34.65) 5.32 (4.95)		UHR group: Mean age = 18.78 (4.93) M/F ratio = 22/28 Employed or studying = 72% SOFAS = 53.12 (8.51) BPRS = 49.43 (8.13) CAARMS = 23.86 (6.43) SANS = 19.42 (14.32) FEP group: Mean age = 19.87 (3.25) M/F ratio = 18/21 Employed or studying = 56% SOFAS = 52.27 (11.56) BPRS = 51.81 (13.50) CAARMS = 32.26 (5.50) SANS = 22.41 (16.51) HC group: Mean age = 21.09 (1.85) M/F ratio = 10/24 Employed or studying = 91% SOFAS = 79.06 (6.96) BPRS = 25.59 (2.46) CAARMS = 3.03 (3.79) SANS = 1.94 (5.37)	Moderate/high	Moderate	Source monitoring with study group as an interaction term, explained 39.8% of variance in EASE scores Aberrant salience explained 6% of variance in EASE scores Aberrant salience was correlated mores strongly with psychopathology measures than EASE scores
Nilsson et al.^32^	2020	Multi-center, Denmark Outpatient unit	ICD-10	EASE	SPD ASD	29 22 51	25.24 (6.42) 7.36 (3.49)		SPD group: Mean age = 23.2 (2.46) M/F ratio = 15/14 Mental problems before age 16 Y/N = 19/8 Mean years of education = 13.7 (2.69) Special needs school Y/N = 5/22 ASD group: Mean age = 23.1 (3.99) M/F ratio = 17/5 Mental problems before age 16 Y/N = 19/1 Mean years of education = 13.7 (2.61) Special needs school Y/N = 16/5	Moderate/high	Moderate	With age, gender, education years, mental health problems and special needs school attendance, there was a statistically significant difference in self-disorders between groups. Self-disorders were higher in SPD group Both groups overlapped in SCAN-related symptoms Self-disorders present a supplementary clinical differentiation between ASD and SPD
Nordgaard et al.^33^	2020	Copenhagen, Denmark Inpatient unit	DSM-IV OPCRIT	EASE	FRS No FRS	30 68 98	20.70 (9.45) 13.20 (7.80)	1.56 (1.10–2.21) *p* = 0.012 1.53 (0.90–2.60) *p* > 0.05	In FRS group: 27 with SZ and 3 with SPD at baseline 30 with SZ at follow up M/F ratio = 9/21 Mean age = 27 In non-FRS group: Participants had SZ, other NAPs, SPD, affective disorders, anxiety, OCD and personality disorders M/F ratio = 24/44 Mean age = 28	Moderate	Moderate	EASE scores higher in FRS group compared to non-FRS group. Also, a moderate correlation between FRS and self-disorders with high significance In whole sample: Odds ratio of 1.56 for having FRSs for each 5-point increase in EASE. Odds ratio associating total EASE with FRS was 1.09 and statistically significant In no FRS sample: Odds ratio of 1.53 for having FRS conversion for each 5-point increase in EASE. Odds ratio associating total EASE with FRS conversion was 1.09 and not statistically significant No FRS in absence of self-disorders Supports the Schneiderian concept of FRS
Nordgaard and Parnas^24^	2014	Copenhagen, Denmark Inpatient unit	ICD-10 DSM-IV OPCRIT	BSABS EASE	NAP SPD OMI	46 22 32 100	19.63 (8.39) 17.82 (6.82) 8.06 (5.89)		Total sample: Mean age = 27.7 M/F ratio = 34/66 Age at first symptom = 19.5 Unmarried = 52% Education -1 school = 40 -High school = 37 -College = 8 -Start uni = 9 -End uni = 6 Unemployed at onset = 20% NAP group: Mean age = 26.5 years M/F ratio = 17/29 Age at first symptom = 16.2 Unmarried = 52% Education -1 school = 52.5% -High school = 35% -College = 37.5% -Start uni = 44.5% -End uni = 83% Unemployed at onset = 22% SPD group: Mean age = 25.0 M/F ratio = 4/18 Age at first symptom = 18.5 Unmarried = 64% Education -1 school = 20% -High school = 22% -College = 12.5% -Start uni = 44.5% -End uni = 17% Unemployed at onset = 9% OMI group: Mean age = 31.22 M/F ratio = 13/19 Age at first symptom = 24.9 Unmarried = 44% Education -1 school = 27.5% -High school = 43% -College = 50% -Start uni = 11% -End uni = 0% Unemployed at onset = 25%	Moderate/high	Moderate	SZ, SPD and other SS groups showed greater aggregation of self-disorders. There were no differences in self-disorders between SZ and SPD, in each domain EASE scores showed moderate correlation with canonical psychopathological dimensions of schizophrenia on symptom scales EASE showed excellent internal consistency
Parnas et al.^34^	2003	Copenhagen, Denmark Outpatient unit	DSM-IV OPCRIT	BSABS	SZ BD	21 23 44	1.47 (1.17) 0.55 (0.94)	**Univariate** 9.07 (2.31–35.65) p 0.00007 **Multivariate** 5.61 (1.21–26.05) p 0.024	SZ group: mean age 33.9; mean duration of illness 9.4 years; medication lifetime 21 (antipsychotic), 3 (lithium), 7 (antidepressants) Bipolar group mean age 45.5, mean duration of illness 15.1 years, medication lifetime 22 (antipsychotic), 19 (lithium), 21 (antidepressants)	Moderate	Low	Participants with an SSD diagnosis scored higher in the domains of perplexity, perception disorders, self-disorders, and cognition disorders, compared to those with BDs. This suggests an aggregation of certain ASEs in SSDs, particularly in the prodromal phases of schizophrenia spectrum psychosis
Parnas et al.^5^	2005	Copenhagen, Denmark Inpatient unit	ICD-10 OPCRIT	BSABS	NAP SPD OMI	51 50 50 151		**SPD** 1.00 (1–1) *p* < 0.05 **NAP vs SPD** 0.74 (0.32–1.72) *p* > 0.05 **NA***p* **+ SPD vs OMI** 3.50 (1.38–8.90) *p* < 0.05	Recruited participants were under 40 years old. They were predominantly female (since aggressive and abusive patients, which tended to be male, were excluded) The OMI group included participants without affective psychosis (e.g.non-psychotic depression)	Moderate	Low	Participants with NAP (including SZ) and SPD scored similarly on scales directed at ASEs, suggesting a common phenomenological picture within SSDs The ASE scale scores were significantly higher in SSDs compared to OMIs
Parnas et al.^35^	2011	Copenhagen, Denmark Inpatient unit	OPCRIT ICD-10	BSABS	SZ + NAP SPD OMI	51 50 50 151	9.59 (6.11) 9.40 (4.80) 4.20 (4.20)	12.00 (2.15–67.07) p 0.003 in those with diagnostic conversion	SZ/psychosis group: Mean age = 25.3 (5.0) M/F ratio = 26/25 Mean duration of illness (m) = 54.6 (59.2) Mean duration of untreated psychosis (m) = 27.3 (42.9) PANSS score -pos = 19.06 (5.8) -neg = 16.95 (6.06) Formal thought disorder = 4.31 (3.07) Anxiety/affective symptoms = 5.91 (3.60) Perplexity = 5.27 (4.39) Perceptual disorder = 2.99 (3.41) SPD group: Mean age = 24.6 (4.4) M/F ratio = 14/36 Mean duration of illness (m) = 84.4 (60.9) Mean duration of untreated psychosis (m) = nil PANSS score -pos = 11.9 (3.1) -neg = 13.3 (4.0) Formal thought disorder = 2.8 (2.3) Anxiety/affective symptoms = 8.6 (3.2) Perplexity = 5.63 (3.3) Perceptual disorder = 2.6 (3.0) OMI group: Mean age = 26.2 (4.6) M/F ratio = 17/33 Mean duration of illness (m) = 90.8 (77.7) Mean duration of untreated psychosis (m) = nil PANSS score -pos = 9.1 (2.3) -neg = 9.7 (3.3) Formal thought disorder = 1.0 (1.5) Anxiety/affective symptoms = 7.8 (3.3) Perplexity = 2.4 (3.1) Perceptual disorder = 2.6 (3.0)	Low	Low	37% of participants (14) had developed a SS diagnosis at follow-up The best predictors for development of SS diagnosis were high levels of perplexity and self-disorder baseline scores No psychological predictor was associated with an escalation within the spectrum Some change in diagnosis but most SZ and SPD patients stable
Raballo et al.^20^	2011	Copenhagen, Denmark Outpatient unit	DSM-III	BSABS	SZ SPD OMI HC	29 61 112 103 305		20.97 (6.82–64.46) *p* < 0.01 11.12 (5.14–24.06) *p* < 0.01 2.60 (1.38–4.87) p 0.03 1 *p* < 0.01	SZ group: Mean age = 43.6 (18.9) M/F ratio = 5/24 Mean duration of illness = 15.5 (13.4) years SPD group: Mean age = 36.4 (14.7) M/F ratio = 29/32 Mean duration of illness = 21.5 (14.7) years OMI group: Mean age = 39.4 (15.0) M/F ratio = 50/62 Mean duration of illness = 18.3 (14.3) years HC group: Mean age = 45.2 (17.8) M/F ratio = 57/46 Mean duration of illness = 0 years	Moderate/low	Moderate	Participants with a diagnosis of SZ or SPD had higher levels of self-disorder scores Self-disorder scores were significantly different in the SS groups compared to the NSS groups SS groups also had a significantly greater likelihood of self-disorders and diagnostic severity
Raballo and Maggini^36^	2005	Parma, Italy Outpatient unit	DSM-IV	BSABS	SZ OCD BD	35 28 23 86	1.31 (1.25) 0.86 (1.04) 1.09 (0.95)	1.44 (0.38–5.50) p 0.59 1.26 (0.27–6.01) p 0**.**77	Mood disorder group: Mean age = 40.1 (9.6) Mean duration of illness = 11.2 (7.2) Mean education = 11.9 (3.9) OCD group: Mean age = 33.9 (7.6) Mean duration of illness = 8.5 (7.2) Mean education = 13.2 (4.1) SZ group: Mean age = 35.2 (8.8) Mean duration of illness = 9.1 (7.0) Mean education = 11.3 (3.5)	Low	Moderate	Participants with schizophrenia expressed elevated scores in self-perceived cognitive disorders and abnormal self-centrality Non statistically significant differences between OCD, MD and SZ on other priori scales of BSABS, including self-disorders Lack of predictive power in SD scale, possibly due to methodological differences (limited interview time) and BSABS not capturing full extent of self-disorder experiences
Spark et al.^37^	2021	Melbourne, Australia Inpatient and Outpatient unit	DSM-IV	EASE	**FEP** SS NSS **FEP + UHR** SS NSS **HC**	39 17 22 86 21 65 34 120	102.94 (12.94) 60.90 (27.13) 101.52 (30.43) 59.95 (28.33) 5.32 (4.95)		In the FEP group: SZ spectrum diagnoses included SZ (8) and schizophreniform (9). Non-SZ spectrum diagnoses included mood disorders + psychosis (9) and non-specified psychoses (13) In the UHR group: SZ spectrum diagnoses included paranoid personality (2) and SPD (2). Non-SZ spectrum diagnoses included anxiety disorder (9), mood disorders (30), substance use disorders (2), and no SCID diagnosis (2) For the combined FEP + UHR group: Whole mean age = 19.56 years SS mean age = 18.33 years NSS mean age = 19.95 years Whole M/F ratio = 35/49 (2 missing) SS M/F ratio = 8/13 NSS M/F ratio = 27/36 Whole BPRS score = 50.45 (10.77) SS BPRS score = 56.48 (10.88) NSS BPRS score = 48.51 (10.06) For the HC group: Mean age = 21.09 years M/F ratio = 9/25 BPRS score = 25.59 (2.46)	Moderate	Moderate	In the FEP and combined FEP and UHR groups, SS and NSS groups showed statistically significance differences in basic self-disturbances (*F* = 19.76, *p* < 0.001) (*F* = 23.56, *p* < 0.001) SS groups expressed greater source monitoring deficits Both groups performed similarly in aberrant salience tasks
Svendsen et al.^38^	2020	Multicentre, Norway Inpatient unit	DSM-IV	EASE	SS NSS	35 21 56	14.70 (9.14) 6.80 (5.78)		Mean age = 32.2 (7.4) M/F ratio = 28/22 Sense of coherence = 41.2 (10.6) GAF-S = 57.2 (16.8) GAF-F = 60.4 (16.9) PANSS = 50.7 (13.5) SFS = 107.6 (10.4)	Moderate/low	Moderate	Basic self-disturbances and sense of coherence show a statistically significant negative correlation. High levels of basic self-disturbance correlate with low levels of sense of coherence. Multivariate analysis confirmed that this correlation was not influenced by diagnostics, clinical symptoms, or level of functioning

Total SD score reported as either mean ± SE or median ± IQR. Odds ratios as log odds ± SE. Values are significant if *p* < 0.05.

ASD = autism spectrum disorder, ASE = anomalous self-experience, BD = bipolar disorder, BPRS = brief psychiatric rating scale, BSABS = Bonn scale for the assessment of basic symptoms, CAARMS = comprehensive assessment of at risk mental states, EASE = Examination of anomalous self-experiences, EAWE = examination of anomalous world-experiences, DSM = diagnostic and statistical manual, F = female, FEP = first episode psychosis, FRS = first rank symptoms, GAF = global assessment of functioning, HC = healthy control, ICD = international classification of diseases, M = male, MD = mood disorder, NAP = non-affective psychosis, NSS = non-schizophrenia spectrum, OCD = obsessive compulsive disorder OMI = other mental illness, OPCRIT = operational criteria checklist, PANSS = positive and negative syndrome scale, RR = SANS = scale for the assessment of negative symptoms, SD = self-disorder, SOC = sense of coherence, SOFAS = social and occupational functioning assessment scale, SPD = schizotypal personality disorder, SS = schizophrenia spectrum, SSD = schizophrenia spectrum disorder, SZ = schizophrenia, UHR = ultra-high risk for psychosis, YMRS = young mania rating scale.

Regarding the target population, studies varied in terms of which participants with SSDs were recruited. Eight out of the 15 studies recruited participants with SSDs exclusively, one study used participants with schizotypal personality disorder (SPD), and one study recruited participants with non-affective psychosis (NAP), which included schizophrenia. Five studies recruited participants with an SSD or SPD and one study recruited participants with either SPD or NAP, which included schizophrenia. Only one study recruited participants based upon symptoms rather than diagnosis, recruiting participants with first rank symptoms (FRS) instead.

When combining the samples of all included studies, a population of 810 participants on the schizophrenia spectrum were included. This consisted of 56 participants with an unspecified SSD, 150 participants with schizophrenia, and 262 participants with an NAP that included schizophrenia. It also contained 262 participants with SPD, 50 CHR participants (with SPD), and 30 participants with FRS.

There was significant variation in the comparison groups for each included study. A mixed composition of OMI was the most common comparison group (five studies), followed by HC (three studies). Other comparison groups included participants with no FRS (one study), autism spectrum disorder (ASD) (one study), no SSD (two studies), non-schizophrenic NAP (one study), bipolar disorder (BD) (one study) and obsessive–compulsive disorder (OCD) (one study).

A comparison population of 781 participants without an SSD were included. This included 302 participants with a variety of OMIs, 195 HCs, and 86 participants with no SSD. Smaller numbers of other comparison groups were also included: no FRS (68), BD (67), OCD (28), ASD (22), and non-schizophrenic NAP (13).

Whilst all studies reported either total SD score or the odds ratio of SDs as a primary outcome, secondary outcomes varied between included studies. Most studies reported clinical secondary outcomes, notably the OPCRIT (six studies), PANSS (seven studies) and GAF (six studies). Neurocognitive outcomes (two studies), aberrant salience outcomes (two studies) and EEG neurophysiology outcomes (one study) were other notable secondary outcomes reported in included studies.

### Risk of bias and quality of evidence assessment

The risk of bias and quality of evidence rating for included studies can be found in Table 1, with a detailed breakdown of each rating in Supplementary Table S2. Concerning the quality of evidence, most studies (11) achieved a moderate quality of evidence score. Four of the 15 studies were determined to have a low quality of evidence. None of the included studies were determined to have a high quality of evidence.

Regarding risk of bias, three of the 15 included studies were determined to have a low risk of bias. Two studies were judged to have a low to moderate risk of bias. Nearly half of the studies (seven) were determined to have a moderate risk of bias. Although three studies were judged to have a moderate to high risk of bias, no studies clearly had a high risk of bias.

### Differences in mean self-disorder score between SSD and control groups in studies using the BSABS

**Figure 2:**
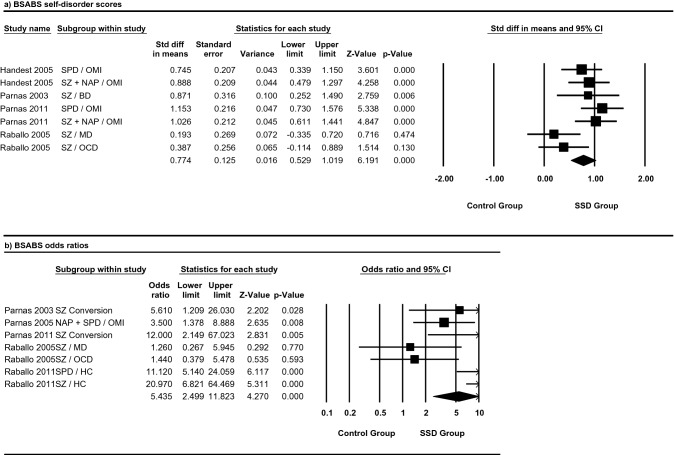
Meta-analysis of the aggregation and likelihood of developing self-disorders in schizophrenia spectrum and control groups, as measured with the Bonn scale for the assessment of basic symptoms (BSABS). Studies are grouped by outcome measure. (**a**) Describes the standard difference in means for total self-disorder score in schizophrenia spectrum and control groups. (**b**) Describes the odds ratios of developing self-disorders in schizophrenia spectrum and control groups. Data shown as mean ± SEM of self-disorder scores for (**a**) and log odds ratio ± SE for (**b**). Both are representative of two independent samples; values are significant if *p* < 0.05. BD = bipolar disorder, HC = healthy control, MD = mood disorder, NAP = non-affective psychosis, OCD = obsessive compulsive disorder, OMI = other mental illness, SD = self-disorder, SPD = schizotypal personality disorder, SZ = schizophrenia.

Panel (a) of Fig. 2 portrays the standardised mean effect sizes and 95% confidence intervals for studies using the BSABS. Three of the four studies (exception^36^) expressed statistically significant effect sizes suggestive of greater SD aggregation in SSD groups compared to control groups. The pooled effect size for BSABS studies was Hedge’s *g* = 0.774, 95% CI 0.529–1.019. The variance for the pooled effect size was Z = 6.191. The pooled effect size was statistically significant (*p* < 0.01). Heterogeneity was moderate (*I*^2^ = 49%).

### The likelihood of expressing self-disorders in SSD versus control groups in studies using the BSABS

Panel (b) of Fig. 2 displays the effect sizes for odds ratios (OR) and the 95% confidence intervals (CI) for studies using the BSABS. The effect sizes of four of the five studies (exception^36^) showed a significantly greater likelihood of SDs in SSD groups compared to controls. The pooled effect size for BSABS studies was OR = 5.435, 95% CI 2.499–11.823. Heterogeneity was judged to be high (*I*^2^ = 66%).

A sensitivity analysis was performed given the high heterogeneity and large variance (> two standard deviations) in three of the studies:^20,]^^4,34,35^^35^. Panels (a) to (d) of Supplementary Fig. S1 describe the odds ratio effect sizes when each potential outlier is removed. Panel (e) of Supplementary Fig. S1 describes the odds ratio effect sizes when all potential outliers are removed. A detailed description of the results from the sensitivity analysis can be found in Supplementary Material 1.

### Differences in mean self-disorder score between SSD and control groups in studies using the EASE with dichotomous scores

**Figure 3:**
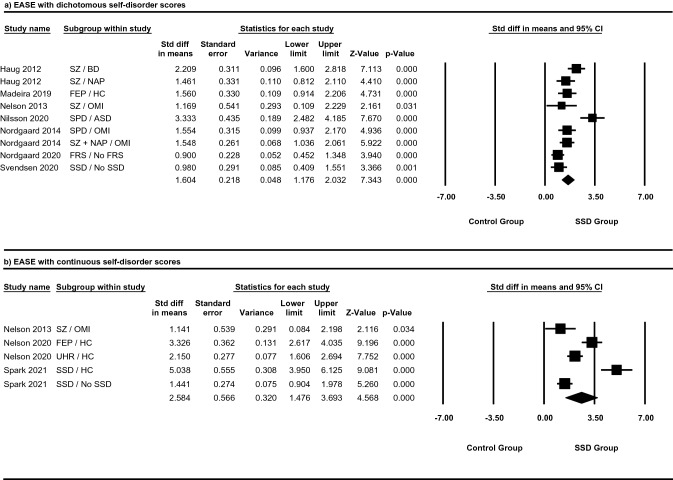
Meta-analysis of the aggregation of self-disorders in schizophrenia spectrum and control groups, as measured with the examination of anomalous self-experiences (EASE). Studies are grouped by the type of self-disorder score reported. (**a**) Describes the standard difference in means for dichotomous total self-disorder scores in schizophrenia spectrum and control groups. (**b**) Describes the standard difference in means for continuous total self-disorder scores in schizophrenia spectrum and control groups. Diamonds indicate pooled effect sizes and squares indicate individual study effect sizes. Data shown as mean ± SEM of self-disorder scores and are representative of two independent samples; values are significant if *p* < 0.05. ASD = autism spectrum disorder, BD = bipolar disorder, FEP = first episode psychosis, FRS = first rank symptoms, HC = healthy control, NAP = non-affective psychosis, OMI = other mental illness, SD = self-disorder, SPD = schizotypal personality disorder, SSD = schizophrenia spectrum disorder, SZ = schizophrenia, UHR = ultra-high risk for psychosis.

Panel (a) of Fig. 3 displays the standardised mean effect sizes and 95% confidence intervals for studies using the EASE with dichotomous scores. The effect sizes for all seven studies showed greater SD scores within SSD groups when compared to control groups. The pooled effect size for EASE studies using dichotomous scores was Hedge’s *g* = 1.604, 95% CI 1.176–2.032. The pooled effect size expressed a variance of Z = 7.343. The pooled effect size was statistically significant (*p* < 0.01). Despite a statistically significant pooled effect size, heterogeneity bordered on very high (*I*^2^ = 76%).

It must be noted that in Nordgaard et al.’s 2020 study on FRS^33^, some patients with schizophrenia but no FRS have been included as controls; as such it would be inaccurate to assume that there were no patients with schizophrenia in the control group. To deal with this issue, we did an additional sensitivity analysis removing this study (also see “Methods” section for three-level analysis for dependent effect sizes). The pooled effect size for EASE studies using dichotomous scores after removing the FRS study was Hedge’s *g* = 1.707, 95% CI 1.266–2.148. The pooled effect size expressed a variance of Z = 7.591. The pooled effect size was statistically significant (*p* < 0.01) and heterogeneity still bordered on very high (*I*^2^ = 72%).

### Differences in mean self-disorder score between SSD and control groups in studies using the EASE with continuous scores

Panel (b) of Fig. 3 presents the standardised mean effect size and 95% confidence intervals for studies using the EASE with continuous scores. All three studies demonstrated effect sizes suggestive of greater SD scores in SSD groups compared to control groups. The pooled effect size for these studies was Hedge’s *g* = 2.584, 95% CI 1.476–3.693. The variance for the pooled effect size was Z = 4.568. The pooled effect size was statistically significant (*p* < 0.01). However, one study^16^ had an effect size which was not significant at the 1% level, although it was significant at the 5% level. Heterogeneity was very high (*I*^2^ = 92%).

### Three-level analysis of combined odds ratio and Hedge’s g effect sizes

In a three-level random-effects model meta-analysis combining all the effect sizes where odds ratios were log-transformed to approximate a normal distribution similar to Hedge’s *g*, the Q-statistic on testing the homogeneity of effect sizes was 94.514 (*p* < 0.001). The estimated heterogeneity at level 2 Tau-squared and at level 3 Tau-squared were 0.5908 and 0.8039, respectively. The level 2 I-squared and the level 3 I-squared were 0.4028 and 0.5481, respectively. SSD status (level 2) and cluster of studies (level 3) explain about 40% and 55% of the total variation, respectively. The average population effect (Z-statistic and its 95% Wald CI) was 2.1429 (1.0915–3.1942).

## Discussion

This meta-analysis is among the first to explore the merit of theories which posit that SDs show a specificity within the schizophrenia spectrum, a finding that is consistent with that from two very recent previous reviews^26,27^. Our meta-analysis appears to indicate a significant magnitude of effect suggestive of a greater expression of SDs within the schizophrenia spectrum population, when compared with HCs and OMIs. This magnitude of effect was observed in both studies using the BSABS and the EASE. Thus, we have found good evidence to support the over-expression of these SD phenomenon within the schizophrenia spectrum, whether they are interpreted as a subgroup of basic symptoms or a more pervasive distortion in the minimal self^7,14,39^.

This overexpression of SDs within the schizophrenia spectrum is further supported by our meta-analysis of odds ratios for the likelihood of SDs occurring. This meta-analysis reported a 2.5–12 times greater likelihood of SDs occurring within the schizophrenia spectrum population, when compared with non-schizophrenia spectrum populations. Even following the removal of outliers, SDs were over one to 4.5 times more likely to occur within schizophrenia spectrum populations when compared to non-schizophrenia spectrum populations.

Despite good evidence suggesting that SDs are a core clinical feature of the schizophrenia spectrum, there are some limitations to the evidence. The variation in pooled effect sizes suggests that SDs are not experienced by everyone within the schizophrenia spectrum. Given that our meta-analysis did not subgroup for different comparator groups, it is difficult to establish the boundaries of SDs in SSDs. This is perhaps reflected in the significant heterogeneity observed across all pooled effect sizes. Alongside methodological differences and variability in target population, the range of different comparison groups likely contributed to this generally high heterogeneity. With high heterogeneity, this study has less confidence in its pooled effect sizes. Also, these results should be interpreted with caution given the results of the three-level meta-analysis. The three-level meta-analysis found effect sizes generated by the meta-analysis to be highly dependent. Another methodological consideration that must be borne in mind is the inclusion of the same patients as separate samples in different types of analyses. Whilst we do not consider it at all likely that this approach is an intrinsic deficiency or a source of significant bias with regard to our findings, we must interpret these results with some caution given the high degrees of variability and inconsistency in the included studies’ original methodologies, which were what necessitated our analytical approach in the first place.

We anticipated that there would likely be some differences in the patterns of results from the EASE and the BSABS from the outset, given their conceptual and methodological differences. In particular, we expected results from BSABS studies to demonstrate less variance than those from EASE studies. Hence, we chose to analyse the BSABS and EASE separately, which has enabled this systematic review to empirically compare the two assessment tools. There are, of course, important caveats with regard to making this comparison. Studies utilising the BSABS have often not used the full scale, and some components of the BSABS (e.g. perceptual disorders) cannot be rated by the EASE and vice versa. However, there are still significant overlaps as to what these two scales measure, which focus on SDs albeit from different schools of thought. The BSABS was designed to facilitate the prediction of imminent risk of psychosis, hence its empirical grounding^7^. This is reflected in the results of our meta-analysis. For studies using the BSABS, we observed a medium to large effect size suggesting greater SD aggregation in SSDs, less variance compared to the EASE, and moderate heterogeneity. The BSABS was developed from the unpublished Heidelberg checklist using in-depth interviews to identify basic symptoms, which were then grouped by clinical reasoning^7,40,41^. The smaller range of items, refined incrementally with a good empirical basis is a likely explanation for these results.

In contrast, the EASE was developed with a focus on exploring the nature and experience of SDs as a core phenotype within the broad schizophrenia spectrum based on self-descriptions obtained from patients suffering from SSDs, thus it has a more theoretical grounding and is informed by the Husserlian approach to phenomenology^7,19^. Studies using the EASE showed a very large effect size suggestive of greater SD aggregation within SSDs, greater variance, and very high heterogeneity. The EASE was developed from a subgroup of BSABS items which were hybridised with philosophical concepts and qualitative explorations of the abnormal self-experiences of those with SSDs^19,41^. Therefore, it is logical that studies using the EASE, with items assessing a greater range of SDs but with less empirical grounding, would express a greater effect size with more variance. It is important to recognise these conceptual differences as they may explain some of the differences observed in the BSABS and EASE study results.

This study gives validation to the concept of SDs as a core clinical feature of the broad schizophrenia spectrum^3^. Therefore, this review hopes to encourage clinicians' interest in phenomenology, since there are vital and clinically relevant findings to be drawn from it. From the perspective of the ipseity disturbance model, the magnitude of effect observed within this analysis gives credit to the concept of SDs as a core clinical vulnerability phenotype of the schizophrenia spectrum, thus informing the construct validity of SSDs. From the perspective of the perceptual anomalies model, this study’s lack of focus on CHR groups prevents commenting on the BSABS’s use in predicting conversion to psychosis. However, the model is supported by the observed effect sizes for SD score and significant odds of SDs being present within SSDs. Regardless of the favoured model, it is apparent that SDs are a core phenomenon within SSDs. These findings could improve clinicians’ understanding of the lived experience of individuals on the schizophrenia spectrum, enabling them to improve patients’ quality of life. From a research perspective, exploration of SDs via the BSABS and the EASE provides one of the most promising avenues for advancing current understanding of psychosis development.

In addition, with these findings on the validity of SD assessment tools in identifying SDs within SSDs, this study hopes to encourage the adoption of assessment tools for SDs within clinical practice. However, we do not recommend the implementation of current assessment tools. Despite the high interrater reliability of the EASE and BSABS^12^^,^^42^, they are lengthy and resource intensive assessments^7^^,^^43^. This creates a difficult conundrum in clinical practice, where the volume of first-hand personal data gathered must be balanced with the limited time clinicians have to perform assessments. This review proposes the development of a shorter assessment than the current BSABS and EASE, aimed at capturing key SD manifestations. What classifies as a key SD phenomenon is beyond the scope of this review but should be investigated. However, this review would like to emphasise that tick-box checklists should not necessarily be pursued for clinical use when assessing SD in patients; their utility is perhaps better suited for the purpose of screening large, potentially healthy, populations for SD. Self-report assessments, such as the FCQ and IPASE, have been shown to be unreliable when used as an SD assessment tool despite their potential as screening measures for SD^43–46^. Both have poor agreement with interviewer assessments, frequently overestimating the presence of SDs. Although there are many valid and reliable tick-box checklists, in the context of SDs, it is a logical extrapolation that tick-box checklists would have the same unreliability in this area. Perhaps future research into SD assessment tools should consider a mixed-methods approach, not unlike the EASE. However, a greater emphasis should be placed on empirically based items, as with the BSABS.

Finally, this study recommends that future research into SDs adopt a more robust methodological framework with more consistent reporting. This recommendation is based on the generally high heterogeneity and inconsistent quality of included studies. At least one study made inappropriate use of statistics, for example using Fisher’s exact to calculate odds ratios in small sample sizes. Often a lower quality was due to bias introduced by a lack of random selection, failure to blind participants and assessors, and samples unrepresentative of the target population. It is worth noting it would be difficult to reduce this bias in some of these studies. Current SD assessments require intense interviewing, making it difficult to introduce blinding. This also makes it difficult to recruit participants with poor cognitive functioning or aggression, an underrepresented subpopulation in included studies. However, if future research can form a standardised protocol for the exploration of SD phenomenon and transparently report methodology, then the reliability of both individual and pooled study results will be improved.

The pooled results of our meta-analysis provide more powerful evidence for the association of SDs and the schizophrenia spectrum than existing individual studies. The findings of this meta-analysis echo the findings of the recent meta-analysis by Raballo et al.^26^. This meta-analysis demonstrated greater effect sizes than this meta-analysis, however this can likely be explained by the greater number of included studies, which in turn increases their analysis’ power. This meta-analysis included fewer studies as our eligibility criteria excluded studies involving adolescent and CHR groups. By contrast, the current analysis included longitudinal studies and performed a three-level meta-analysis with nested effect sizes. This analysis showed a high level of dependency and so reduces the confidence with which conclusions can be drawn. However, by performing it the robustness of this meta-analysis’ methodology and the reliability of its results have been increased.

Whilst not the first published meta-analysis within this field^26,27^, this meta-analysis is the first to opt to analyse the BSABS and EASE separately. Although this reduces the power of pooled effect sizes, it improves the precision of the results and allows for more accurate conclusions to be drawn. It also facilitates comparison of each assessment tool.

However, there are several limitations to this study. Firstly, the meta-analysis did not perform subgroup analysis on different comparison groups. HCs and OMIs as comparisons provide their own respective strengths and weaknesses. This study chose not to perform the subgroup analysis to maintain sample sizes and power. However, this likely accounted for a proportion of observed heterogeneity.

This meta-analysis originally intended to calculate the prevalence of SDs in SSDs. However, the lack of a cut-off score for the presence/absence of an SD prevented this from being done. Although this was compensated for by pooling odds ratios for the likelihood of SDs, it still diminishes the accuracy and generalisability of our results.

Finally, this meta-analysis chose not to explore populations with a CHR for SSDs. This was done given the sometimes fleeting and non-specific nature of SDs within CHR populations, which would not warrant accurate measurement of SDs. However, the use of these assessment tools in the prediction of SSD conversion risk is a major area of interest for their clinical utility. Therefore, by not analysing this population, this study cannot make a complete assessment of the clinical utility of these SD assessment tools.

In summary, evidence from this meta-analysis suggests that SDs show a greater aggregation and likelihood of occurring within the broad schizophrenia spectrum, when compared to HCs and OMIs. This aids in validating SDs as a core clinical feature of SSDs, which carries implications for aetiological research into SSDs. Assessment tools for SDs have potential for clinical application, however, this might be unlikely in their current iterations.

## Methods

### Search strategy and data collection

This systematic review was conducted following the guidelines provided in the Preferred Reporting Items for Systematic Reviews (PRISMA) statement checklist^47^. The electronic literature search was conducted by one researcher (S.B.) using the databases Medline, Embase, PsychINFO, PubMed and the Web of Science. To ensure all relevant literature was captured, grey literature was searched for on Google Scholar, Opengrey, Proquest, and Psychextra. The references and citations of included studies were also explored to gather literature missed in the initial search. Where relevant, the researchers contacted the authors of identified studies with inaccessible, incomplete, or ongoing trials to gather extra data.

In line with other systematic reviews, a condition, context, and population (CoCoPop) process was developed for this systematic review and was as follows: the condition as adults with an SD; the context as any setting; and the population as adults with a clinically diagnosed SSD or first episode non-affective psychosis (NAP).

A detailed description of the methodology for data collection can be found in Supplementary Materials. This includes rationales for the search terms, the screening process, the review process, and the process for handling disputes. Supplementary Material 3 presents an example search strategy. The eligibility criteria for included studies are shown in Supplementary Table S1. To summarise, the following inclusion criteria were applied: participants with a diagnosis of SSD or NAP only, inclusion of an SD assessment tool, inclusion of an observer rated SD score, adult only participants (mean age > 18 years old), English language only, participants with a clinical diagnosis only, and inclusion of a comparison group. The following exclusion criteria were applied: publication pre-1967, inclusion of a self-reported SD score, inclusion of children or adolescents, non-English language, participants with research diagnoses only, no comparison group, single case studies, and qualitative studies.

### Data extraction

The researcher (S.B.) responsible for data collection carried out data extraction in line with the PRISMA statement guidelines^47^. The research supervisor (C.H.) provided oversight to the process for quality assurance. The full texts of included articles were re-read, with key characteristics extracted and placed within a summary of findings table (Table 1). Key characteristics reported within the table included geographical setting, instrument for clinical diagnosis, SD assessment tool, sample type, sample size, SD assessment results (mean SD score), SD odds ratio, key findings, descriptive psychopathology, and demographic features of the sample. The summary of findings table included a rating for “quality of evidence” and “risk of bias”. To maintain transparency, a detailed summary of how each quality of evidence and risk of bias rating was determined can be found in Supplementary Table S2.

A detailed description of the methodology for quality of evidence and risk of bias assessments is given in Supplementary Materials. The quality of included studies was determined through assessment with the Grading of Recommendations, Assessment, Development, and Evaluation (GRADE) handbook^48^ (Supplementary Materials). Study quality was graded from “high” to “very low”. The risk of bias for included studies was determined using a risk-of-bias tool designed specifically for systematic reviews of prevalence studies (Supplementary Materials)^49^. Study bias was rated from “low” to “high”.

### Data analysis (systematic review and meta-analysis)

A narrative synthesis in line with Cochrane guidelines was performed on the 15 studies which met the aims and eligibility criteria of this systematic review^50,51^. This involved an initial synthesis of data relating to the utility of SD assessment tools, followed by the extraction of relevant findings (Table 1) mentioned above. Findings relevant to the research aims were systematically discussed and both the quality and potential bias in included studies were critically appraised.

A meta-analysis was performed on all included studies with an SSD group and at least one other comparison group. Given the varied comparison groups for different included studies, we anticipated considerable heterogeneity. Meta-analysis was performed using the Comprehensive Meta-analysis Professional statistical software package version 3.0.

For descriptive analysis, all results were reported via a random-effects model^52^. The random-effects model was favoured for the anticipated heterogeneity in methodologies of different included studies in this field of research. The random-effects model’s aim of facilitating inferences about population level effects also aligned with this systematic review’s research aims^52,53^. Effect sizes were calculated for standardised differences (Hedge’s *g* with 95% confidence intervals) in mean total SD scores and the odds ratios of having an SD.

Using Comprehensive Meta-Analysis, heterogeneity was quantified and assessed with Cochran’s *Q* and *I*^2^ statistics^54^. Regarding *I*^2^, heterogeneity was graded as follows: low heterogeneity (0–25%), moderate heterogeneity (25–50%), high heterogeneity (50–75%), and very high heterogeneity (75–100%)^55,56^. Sensitivity analyses were performed on some models with very high heterogeneity. Potential outliers were identified as studies with point estimates over 2 standard deviations and *p* < 0.05. Each potential outlier was removed individually, and the model recalculated to determine their individual impact on the effect size and heterogeneity. Finally, all outliers were removed, and the model recalculated.

Due to the significant differences between assessment with the EASE and BSABS, subgroup analysis was performed on the type of SD assessment tool used. Assessment with the EASE does not enable the generation of odds ratios because there is no quantitative cut-off score for the presence/absence of SDs. Therefore, odds ratios for the presence/absence of SDs were only performed on studies using the BSABS. A further subgroup analysis was performed on EASE studies based off the type of scoring reported (dichotomous or continuous scores).

Some included studies used the same sample of participants as other included studies or were follow-ups of other included studies. For this reason, a three-level random-effects meta-analysis with nested (dependent) effect sizes in R using the metaSEM package was performed^57^. For the purpose of this analysis, relevant studies using the BSABS and EASE were mixed together. This was done given the highly dependent nature of the effect sizes. Odds ratios, such as in the Parnas et al. studies^5,35^, were log-transformed to approximate a normal distribution like Hedge’s *g*. Also, only one set of effect sizes (usually SSD/HC) were selected for each comparison.

Finally, to assess potential publication bias, a funnel plot was produced encompassing all studies included in the current meta-analysis (see Supplementary Materials)^58^.

## Supplementary Information

Supplementary Information.

## References

[1] Parnas J, Henriksen MG (1990). Disordered self in the schizophrenia spectrum: A clinical and research perspective. Harv. Rev. Psychiatry.

[2] National center for biotechnology information MedGen. Schizophrenia spectrum and other psychotic disorders. 2020.

[3] Wright M (2020). Schizophrenia and schizophrenia spectrum disorders. J. Am. Acad. Phys.n Assist..

[4] Parnas J (2011). A disappearing heritage: The clinical core of schizophrenia. Schizophr. Bull..

[5] Parnas J, Handest P, Jansson L, Sæbye D (2005). Anomalous subjective experience among first-admitted schizophrenia spectrum patients: Empirical investigation. Psychopathology.

[6] Schultze-Lutter F (2009). Subjective symptoms of schizophrenia in research and the clinic: The basic symptom concept. Schizophr. Bull..

[7] Schultze-Lutter, F. Michel, C. Flückiger, R. & Theodoridou, A. *Risk Factors For Psychosis Ch. 4* (Academic Press, Cambridge, 2020).

[8] Giersch A, Mishara AL (2017). Is schizophrenia a disorder of consciousness? Experimental and phenomenological support for anomalous unconscious processing. Front. Psychol..

[9] Kaminski, J. Sterzer, P. & Mishara A.L. Seeing rain: Combining phenomenological and bayesian predictive coding approaches to visual hallucinations in schizophrenia. *Conscious. Cogn.* **73,** 102757 (2019). 10.1016/j.concog.2019.05.005 31284176

[10] Mishara AL (2015). Neurobiological models of self disorders in early psychosis. Schizophr. Bull..

[11] Sterzer P, Mishara AL, Voss M, Heinz A (2016). Thought insertion as self disturbance (Ichstörung): An integration of predictive coding and phenomenological approaches. Front. Hum. Neurosci..

[12] Vollmer-Larsen A, Handest P, Parnas J (2007). Reliability of measuring anomalous experience: The Bonn scale for the assessment of basic symptoms. Psychopathology.

[13] Schultze-Lutter F, Addington J, Ruhrmann S, Klosterkötter J (2007). Schizophrenia Proneness Instrument, Adult Version (SPI-A).

[14] Sass LA, Parnas J (2003). Schizophrenia, consciousness, and the self. Schizophr. Bull..

[15] Humpston CS, Broome MR (2020). Thinking, believing, and hallucinating self in schizophrenia. The Lancet Psychiatry..

[16] Nelson B, Thompson A, Chanen AM, Amminger GP, Yung AR (2013). Is basic self-disturbance in ultra-high risk for psychosis (‘prodromal’) patients associated with borderline personality pathology?. Early. Interv. Psychiatry..

[17] Zandersen M, Parnas J (2019). Identity disturbance, feelings of emptiness, and the boundaries of the schizophrenia spectrum. Schizophr. Bull..

[18] Nelson B, Parnas J, Sass LA (2014). Disturbance of minimal self (ipseity) in schizophrenia: clarification and current status. Schizophr. Bull..

[19] Parnas J (2005). EASE: examination of anomalous self-experience. Psychopathology.

[20] Raballo A, Sæbye D, Parnas J (2011). Looking at the schizophrenia spectrum through the prism of self-disorders: An empirical study. Schizophr. Bull..

[21] Henriksen MG, Nordgaard J (2014). Schizophrenia as a disorder of the self. J. Psychopathol..

[22] Mishara AL, Lysaker PH, Schwartz MA (2014). Self-disturbances in schizophrenia: History, phenomenology, and relevant findings from research on metacognition. Schizophr. Bull..

[23] Koren D (2020). Basic self-disorders in adolescence predict schizophrenia spectrum disorders in young adulthood: A 7-year follow-up study among non-psychotic help-seeking adolescents. Schizophr. Res..

[24] Nordgaard J, Parnas J (2014). Self-disorders and the schizophrenia spectrum: A study of 100 first hospital admissions. Schizophr. Bull..

[25] Raballo A, Parnas J (2011). The silent side of the spectrum: Schizotypy and the schizotaxic self. Schizophr. Bull..

[26] Raballo, A. Poletti, M. Preti, A. & Parnas, J. The self in the spectrum: A meta-analysis of the evidence linking basic self-disorders and schizophrenia. *Schizophr. Bull*. **Sbaa201,** (2021). 10.1093/schbul/sbaa201 PMC8266610 33479736

[27] Henriksen MG, Raballo A, Nordgaard J (2021). Self-disorders and psychopathology: A systematic review. Lancet Psychiatry.

[28] Handest P, Parnas J (2005). Clinical characteristics of first-admitted patients with ICD-10 schizotypal disorder. Br. J. Psychiatry..

[29] Haug E (2012). Selective aggregation of self-disorders in first-treatment DSM-IV schizophrenia spectrum disorders. J. Nerv. Ment. Dis..

[30] Madeira, L. e al. Self and world experience in non-affective first episode of psychosis. *Schizophr. Res.* **211,** 69–78 (2019). 10.1016/j.schres.2019.07.001 31307860

[31] Nelson, B. et al. The neurophenomenology of early psychosis: An integrative empirical study. *Conscious. Cogn*. **77,** 102845 (2020). 10.1016/j.concog.2019.102845 31678780

[32] Nilsson M (2020). Self-disorders in asperger syndrome compared to schizotypal disorder: A clinical study. Schizophr. Bull..

[33] Nordgaard, J. Henriksen, M.G. Berge, J. & Nilsson, L.S. (2020). Associations between self-disorders and first-rank symptoms: An empirical study. *Psychopathology.* **53,** 103–110 (2020). 10.1159/000508189 32610320

[34] Parnas J, Handest P, Saebye D, Jansson L (2003). Anomalies of subjective experience in schizophrenia and psychotic bipolar illness. Acta. Psychiatr. Scand..

[35] Parnas J (2011). Self-experience in the early phases of schizophrenia: 5-year follow-up of the Copenhagen prodromal study. World Psychiatry.

[36] Raballo A, Maggini C (2005). Experiential anomalies and self-centrality in schizophrenia. Psychopathology.

[37] Spark J (2021). Distinguishing schizophrenia spectrum from non-spectrum disorders among young patients with first episode psychosis and at high clinical risk: The role of basic self-disturbance and neurocognition. Schizophr. Res..

[38] Svendsen, I. H. et al. Basic self-disturbances are associated with sense of coherence in patients with psychotic disorders. *Plos One*. **15,** e0230956 (2020). 10.1371/journal.pone.0230956 PMC7159222 32294097

[39] Sass LA (2014). Self-disturbance and schizophrenia: Structure, specificity, pathogenesis (current issues, new directions). Schizophr. Res..

[40] Gross, G. Huber, G. Klosterk€otter, J. & Linz, M. 1987. *Bonner Skala f€ur Die Beurteilung Von Basissymptomen (BSABS; Bonn Scale For The Assessment Of Basic Symptoms)* (Springer-Verlag, Berlin, 1987).

[41] Schultze-Lutter F (2016). Revisiting the basic symptom concept: towards translating risk symptoms for psychosis into neurobiological targets. Front. Psychiatry..

[42] Møller P, Haug E, Raballo A, Parnas J, Melle I (2011). Examination of anomalous self-experience in first-episode psychosis: Interrater reliability. Psychopathology.

[43] Parnas, J. Nordgaard, J, & Henriksen, M.G. Panic, self-disorder, and EASE Research: Methodological considerations. *Psychopathology*. **50,** 169–170 (2017). 10.1159/000471505 28395290

[44] Nelson B (2019). The construct validity of the Inventory of Psychotic-Like Anomalous Self-Experiences (IPASE) as a measure of minimal self-disturbance: Preliminary data. Early Interv. Psychiatry..

[45] Maß, R. Hitschfeld, K. Wall, E. & Wagner, H.B. Validit€ at der erfassung schizophrener basissymptome. *Nervenarzt*. **68,** 205e211 (1997). 10.1007/s001150050115 9198780

[46] Michel, C. Kutschal, C. Schimmelmann, B.G. & Schultze-Lutter, F. Convergent and concurrent validity of the Frankfurt Complaint Questionnaire as a screener for psychosis risk. *J. Risk. Res*. **20,** 1480e1496 (2017).

[47] Moher, D. Liberat,i A. Tetzlaff, J. Altman, DG. & The PRISMA Group. Preferred reporting items for systematic reviews and meta-analyses: The PRISMA statement. *PLoS Med*. **6,** e1000097 (2009). PMC3090117 21603045

[48] Guyatt, G. et al. GRADE guidelines: 1. Introduction—GRADE evidence profiles and summary of findings tables*. J. Clin. Epidemiol*. **64,** 383–394 (2011). 10.1016/j.jclinepi.2010.04.026 21195583

[49] Hoy D (2012). Assessing risk of bias in prevalence studies: Modification of an existing tool and evidence of interrater agreement. J. Clin. Epidemiol..

[50] Higgins JP (2019). Cochrane handbook for systematic reviews of interventions.

[51] Ryan R. & Cochrane consumers and communication review group*. ‘Cochrane consumers and communication review group: Data synthesis and analysis’*. (2013). 10.1111/jebm.12066 24325413

[52] Borenstein M, Hedges LV, Higgins JP, Rothstein HR (2010). A basic introduction to fixed-effect and random-effects models for meta-analysis. Res. Synth. Methods..

[53] Hedges L, Vevea J (1998). Fixed- and random-effects models in meta-analysis. Psychol. Methods..

[54] Higgins JP, Thompson SG (2002). Quantifying heterogeneity in a meta-analysis. Stat. Med..

[55] Higgins JP, Thompson SG, Deeks JJ, Altman DG (2003). Measuring inconsistency in meta-analyses. BMJ.

[56] Huedo-Medina TB, Sánchez-Meca J, Marín-Martínez F, Botella J (2006). Assessing heterogeneity in meta-analysis: Q statistic or I2 index?. Psychol. Methods..

[57] Cheung MWL (2019). A guide to conducting a meta-analysis with non-independent effect sizes. Neuropsychol. Rev..

[58] Duval S, Tweedie R (2000). Trim and fill: A simple funnel-plot-based method of testing and adjusting for publication bias in meta-analysis. Biometrics.

