# Supplementary material for: Prevalence and assessment of self-disorders in the schizophrenia spectrum: a systematic review and meta-analysis
Source: Sci Rep. 2022 Jan 21;12:1165. doi: 10.1038/s41598-022-05232-9 (PMC8782935; doi:10.1038/s41598-022-05232-9)
Supplement: Supplementary file 1 — Supplementary Information. [file 41598_2022_5232_MOESM1_ESM.docx]

**Supplementary Information**

**Supplementary Table 1** Table of eligibility criteria for inclusion and exclusion in this systematic review

| **Inclusion criteria** | **Exclusion criteria** |
| --- | --- |
| Any studies with participants who have a defined diagnosis of Schizophrenia Spectrum Disorder (SSD) or non-affective psychosis (NAP) | Any studies pre-1967 |
| Any studies involving the Self-disorder/Self-disturbance and assessment (EASE, SPI, BSABS) | Any studies with self-reported Self-disorders |
| Any studies with an observer rated self-disorder assessment score | Any studies with no observer rated self-disorder assessment score |
| Any adult only studies | Any children or adolescent studies |
| Any English language studies | Any non-English language studies |
| Any studies with participants who have a clinical diagnosis | Any studies with participants who have a research diagnosis only |
| Any studies with a comparison group(s) | Any studies with no comparison group(s) |
|  | Any single case studies |
|  | Any studies with qualitative methods |

Table reporting the inclusion and exclusion criteria for studies assessed during the review phase of data collection. The full text articles of all studies identified during screening were read and assessed against these criteria.

Abbreviations: BSABS = Bonn scale for the assessment of basic symptoms, EASE = examination of anomalous self-experiences, NAP = non-affective psychosis, SD = self-disorder, SPD = schizotypal personality disorder, SPI = schizophrenia proneness instrument, SSD = schizophrenia spectrum disorder.

**Supplementary Table 2** Table of Quality of Evidence & Risk of Bias for included studies.

| **Author (s)** | **Year** | **Risk of bias rating** | **Risk of bias rationale** | **Quality of evidence rating** | **Quality of evidence rationale** |
| --- | --- | --- | --- | --- | --- |
| Handest and Parnas ^[28]^ | 2005 | Low | 1. Yes – Target population is SPD and OMI only so representative of national SSD population. Even more so when consider psychosis group captured SZ, schizoaffective and delusional disorder patients. 2. Yes/no – Target population is the SPD population. The study does not present a table of demographic features of the sample. There were no age differences but only participants under 40 were included. Overall were no stat significant gender differences, but the schizotypal and OMI group had more females than males. Patients with severe psychosis, aggression, or substance abuse were excluded potentially affecting the representation of the sample. Can’t tell if inpatient or outpatient 3. No/can’t tell – 155 consecutively admitted patients were included. There is no mention of random selection 4. Can’t tell – 4 participants were later excluded due to having somatic illness. However, as the demographic characteristics of responders and non-responders are not commented on in detail, non-response bias cannot be determined. 5. Yes – data was collected directly from participants via direct semi-structured interview (3-5h) 6. Yes – case definition was specified as an ICD-10 clinical diagnosis of schizotypal disorder, backed by OPCRIT classification for all group disorders. 7. Yes – the BSABS has proven validity and high interrater reliability 8. Yes – all participants underwent the same semi structured interview and battery of psychopathology assessments 9. Can’t tell – a prevalence period was not specified 10. Yes – need a cut-off score for presence/absence of self-disorder which is possible with BSABS 11. Moderate to high internal validity, moderate external validity = low risk of bias | Moderate | Study limitations/risk of bias   - Some failure to develop and apply appropriate eligibility criteria   - Exclusion criteria of no aggressive or substance abuse participants led to bias in gender of schizotypal group & OMI group   - Otherwise, limited bias here   - No healthy control groups - Some apparent flawed measurement of both exposure and outcome   - No apparent differences in measurement for exposure   - Cross sectional study - No apparent failure to adequately control confounding   - Logistic & multivariate logistic regression performed (mainly ordinal data) to account for confounding - No follow up but all groups followed for same amount of time   Inconsistency of results   - Cannot comment on variance of point estimates - But seems to be no overlap of CIs and all p values < 0.05   Indirectness of evidence   - Population is applicable to the systematic review   - Patients with SPD and NAP - The intervention is applicable to the systematic review   - BSABS as self-disturbance assessment tool - The outcome measures are applicable to the systematic review   - Mean total self-disorder score and SD - No indirect comparison   - OMI   Imprecision   - No apparent imprecision   Publication bias  Magnitude of effect   - OR so cannot convert to RR to calculate if large magnitude of effect   Dose-response curve   - No dose response curve   Residual confounding   - Doesn’t seem to have occurred |
| Haug et al ^[29]^ | 2012 | Moderate | 1. Can’t tell/no – Target population is SZ, BD and OMI only and appears representative of national populations for these respective conditions. In terms of SSD population, only reports SZ and unknown whether other SSDs are captured in the target population so less representative. 2. Yes – Target population is the SZ population. SZ, BD and OMI group well represented in sample. Group sizes not matched. The study presents a table of demographic features of the sample. There were no stat significant differences for age or affective symptoms. Efforts to include patients with substance misuse disorder if not currently using. Can’t tell if inpatient or outpatient but reported as near epidemiological sample 3. Can’t tell/no – there is no mention of random selection. Interviewers were also not blinded but Bonferroni post-hoc tests performed to minimise impact. Analysing statistics were 4. Yes – in follow up study there was no significant differences between responders and non-responders 5. Yes – data was collected directly from participants in semi-structured interviews 6. Yes – case definition as the DSM-IV diagnosis of SZ, BD or OMI and confirmed by research SCID 7. Yes – the EASE has proven validity and high interrater reliability 8. Yes - all participants underwent the same semi structured interview and battery of psychopathology assessments 9. Can’t tell – a prevalence period was not specified 10. No – need a cut-off score for presence/absence of self-disorder which is not possible with EASE 11. Moderate internal validity and moderate external validity = moderate risk of bias | Moderate | Study limitations/risk of bias   - Minor failures to develop and apply appropriate eligibility criteria   - Eligibility criteria appropriately applied but participants which did not fully meet original criteria were added to increase statistical power. Were still from the same setting   - No healthy control groups   - Managed to include patients with previous substance disorders by allowing patients with history of substance abuse but no recent use - Some flawed measurement of both exposure and outcome   - No outcome differences between group   - No assessment blinding – necessary to facilitate interview process – Bonferroni’s post-hoc tests to minimise impact of this on EASE scores   - Cross sectional study - No apparent failure to adequately control confounding   - Range of demographic features were reported and presented – only differences were in duration of psychosis, psychotic and global psychopathology functioning - No apparent o inadequately short follow-up   - Cross sectional so no need for follow-up   Inconsistency of results   - Cannot comment on variance of point estimates - Overlap of confidence intervals only for age, sex, duration of psychosis, GAF function, PANSS pos & neg scores, YMRS score, and CDSS score – these recognised by author in general logistic regression and deemed not correlated with SZ diagnosis, unlike SD measured by EASE - All EASE scores statistically significant and only predictor (SPD diagnosis) of EASE total scores, adjusted ORs for SZ vs BD and SZ vs OMI showed p <0.05 and no CI overlap.   Indirectness of evidence   - Population is applicable to the systematic review   - Patients with SZ, BD, and OMI - The intervention is applicable to the systematic review   - EASE as self-disturbance assessment tool - The outcome measures are applicable to the systematic review   - Mean total self-disorder score and SD, OR - No indirect comparison   - BD and OMI   Imprecision   - No apparent imprecision   Publication bias  Magnitude of effect   - No RR to calculate   Dose-response curve   - No dose-response curve   Residual confounding |
| Madeira et al ^[30]^ | 2019 | Moderate | 1. Yes/no – Target population is First episode NAP so not representative of the entire national population. Non-affective psychosis should capture all SSDs but may also include other non-SS conditions. 2. Yes/no – Target population is the first episode NAP population. The study presents a table of demographic features of the sample. Participants were matched for all demographic features with the healthy controls except for education. Patients with mood disorders and psychosis, substance misuse disorder, psychiatric comorbidity, and brain disorders were excluded potentially affecting the representation of the sample. But substance use was included. But hospital patients so less generalisable 3. Can’t tell/no – There is no mention of random selection 4. Can’t tell – no apparent nonresponse bias as no dropouts 5. Yes – data was collected directly from participants via direct semi-structured interview 6. Yes – case definition was specified as an DSM-IV clinical diagnosis of NAP 7. Yes – the EASE has proven validity and high interrater reliability 8. Yes – all participants underwent the same semi structured interview and battery of psychopathology assessments 9. Can’t tell – a prevalence period was not specified 10. No – need a cut-off score for presence/absence of self-disorder which is not possible with EASE 11. Moderate internal validity, moderate external validity = moderate risk of bias | Moderate | Study limitations/risk of bias   - No apparent failures to develop and apply appropriate eligibility criteria   - Eligibility criteria appropriately applied, and population and control were well matched in demographic criteria – only education and   - Healthy control group   - Participants with severe substance misuse disorders, FEP mood disorders, brain disorders and psychiatric comorbidity were excluded – not if had use substances or alcohol though - No apparent flawed measurement of both exposure and outcome   - No outcome differences between group   - There was clinical blinding   - Cross sectional study - No apparent failure to adequately control confounding   - Range of demographic features were reported and presented – only differences were in education – otherwise baseline characteristics matched - No apparent inadequately short follow-up   - Cross sectional so no need for follow-up   Inconsistency of results   - Significant variance in point estimates for mean EASE and EAWE scores - No overlap of CI for correlation between EASE and EAWE - All EASE scores statistically significant and only predictor (SPD diagnosis) of EASE total scores, adjusted ORs for SZ vs BD and SZ vs OMI showed p <0.05 and no CI overlap.   Indirectness of evidence   - Population is applicable to the systematic review   - Patients with first episode NAP - The intervention is applicable to the systematic review   - EASE as self-disturbance assessment tool - The outcome measures are applicable to the systematic review   - Mean total self-disorder score (EASE), mean total EAWE score, correlation - No indirect comparison   - HC   Imprecision   - No apparent imprecision   Publication bias  Magnitude of effect   - No RR to calculate   Dose-response curve   - No dose-response curve   Residual confounding |
| Nelson et al ^[16]^ | 2013 | Moderate | 1. Can’t tell/yes – Target population is FEP and UHR so appears representative of the national population. In terms of SSD population representation, not stated which SSDs so specifics of representativeness unknown but still participants with SSDs were explored as a separate sub sample so representative. 2. Yes/no - Target population is the FEP SZ population. The study presents a table of demographic features of the sample. Participants were matched for all demographic features with the healthy controls except for education. Participants with IQ <70, lack of proficiency with English, organic brain disorder, and participants aged 15-25 were excluded potentially affecting the representation of the sample. Outpatient so more generalisable 3. Can’t tell/no – No mention of random selection 4. Can’t tell – no apparent nonresponse bias as no dropouts 5. Yes – data was collected directly from participants via direct semi-structured interview 6. Yes – case definition was specified for FEP was daily frank positive psychotic symptoms for longer than one week 7. Yes – the EASE has proven validity and high interrater reliability 8. Yes – all participants underwent the same semi structured interview and battery of psychopathology assessments 9. Can’t tell – a prevalence period was not specified 10. No – need a cut-off score for presence/absence of self-disorder which is not possible with EASE 11. Moderate internal validity, moderate external validity = moderate risk of bias | Low | Study limitations/risk of bias   - Some failures to develop and apply appropriate eligibility criteria   - Eligibility criteria appropriately applied   - Healthy control group   - Participants who were older than 15-25, organic brain disorder, IQ <70 and not fluent in English were excluded – may introduce bias   - Groups did not differ and so were likely matched on all but education (lower for SZ group) - Possibly some flawed measurement of both exposure and outcome   - No outcome differences between group   - No mention of interviewer blinding   - Cross sectional study/case series - No apparent failure to adequately control confounding   - Range of demographic features were reported and presented – only differences were in education – otherwise baseline characteristics matched   - Unknown if all relevant confounders captured   - Multiple regression for confounding was not performed - No apparent inadequately short follow-up   - No follow up at this point – would be reported in subsequent study   Inconsistency of results   - Seemed to be significant variance for point estimates of total and some domain EASE scores for SZ group and other psychosis group - CI not reported for many statistics - P values of t tests not reported   Indirectness of evidence   - Population is applicable to the systematic review   - Patients with SZ spectrum disorder in first episode - The intervention is applicable to the systematic review   - EASE as self-disturbance assessment tool - The outcome measures are applicable to the systematic review   - Mean total self-disorder score (EASE) - No indirect comparison   - Other psychosis in first episode   Imprecision   - Possibly some imprecision but cannot say off reported statistics   Publication bias  Magnitude of effect   - No RR to calculate   Dose-response curve   - No dose-response curve   Residual confounding |
| Nelson et al ^[31]^ | 2020 | Moderate / High | 1. No – Target population is FEP and UHR so not representative of the entire national population. In terms of capturing SSD population, FEP group included different SSDs but also had some mood disorders and anxiety disorders so less representative 2. Yes/no - Target population is the FEP and UHR population. The study presents a table of demographic features of the sample. Participants were matched for all demographic features with the healthy controls except for education. Participants with IQ <70, lack of proficiency with English and participants aged 15-25 were excluded potentially affecting the representation of the sample. Outpatient so more generalisable 3. Can’t tell/no – No mention of random selection 4. Can’t tell – no apparent nonresponse bias as no dropouts 5. Yes – data was collected directly from participants via direct semi-structured interview 6. Yes – case definition was specified for UHR as attenuated psychotic symptoms, brief limited intermittent psychotic symptoms, trait vulnerability and deteriorating or chronic low functioning; and for FEP daily positive psychotic symptoms for longer than one week 7. Yes – the EASE has proven validity and high interrater reliability 8. Yes – all participants underwent the same semi structured interview and battery of psychopathology assessments 9. Can’t tell – a prevalence period was not specified 10. No – need a cut-off score for presence/absence of self-disorder which is not possible with EASE 11. Moderate internal validity, low external validity = moderate/high risk of bias | Moderate | Study limitations/risk of bias   - Some failures to develop and apply appropriate eligibility criteria   - Eligibility criteria appropriately applied   - Healthy control group but no clinical control group   - Participants who were older than 15-25, IQ <70 and not fluent in English were excluded – may introduce bias   - Groups were not clearly matched for age, employment/study and psychopathology scores - Possibly some flawed measurement of both exposure and outcome   - No outcome differences between group   - No mention of interviewer blinding   - Cross sectional study - No apparent failure to adequately control confounding   - Range of demographic features were reported and presented – only differences were in education – otherwise baseline characteristics matched   - Unknown if all relevant confounders captured   - Multiple regression was performed to adjust for confounding - No apparent inadequately short follow-up   - No follow up at this point – would be reported in subsequent study   Inconsistency of results   - Significant variance in point estimates for mean EASE and other recorded outcome measure scores - CI not reported for many statistics - All EASE scores statistically significant. Other measures such as aberrant salience, etc do not have had statistically insignificant scores. Especially with multiple regression and correlations   Indirectness of evidence   - Population is applicable to the systematic review   - Patients with FEP and UHR for SZ - The intervention is applicable to the systematic review   - EASE as self-disturbance assessment tool - The outcome measures are applicable to the systematic review   - Mean total self-disorder score (EASE), other neurophenomenological measures (such as for aberrant salience), correlation - No indirect comparison   - HC   Imprecision   - No apparent imprecision   Publication bias  Magnitude of effect   - No RR to calculate   Dose-response curve   - No dose-response curve   Residual confounding |
| Nilsson et al ^[32]^ | 2020 | Moderate/ High | 1. No – Target population is SPD and ASD only and appears representative of SPD and ASD national population. In relation to capturing SSD national population, SPD does not encompass all of SZ spectrum disorders 2. No – Target population is the SPD and ASD population. The sample is relatively representative of the target population, however the inclusion and exclusion criteria introduced selection bias by giving only a high functioning and well-demarcated sample. Outpatient so less generalisable 3. No – there is no mention of random selection. Care is taken to ensure clearly diagnosed patients with a single diagnosis are recruited as well, further limiting random selection. Interviewers are also not blinded. 4. Can’t tell - The demographic characteristics of patients later excluded from the statistical analysis were not given, therefore it is hard to comment on non-response bias. Given they were not included however, and there was no dropouts, likely minimal non-response bias 5. Yes – data was collected directly from participants in semi-structured interviews 6. Yes – a case definition for ASD and SPD was selected using ICD-10 diagnostic criteria 7. Yes – the EASE has proven validity and high interrater reliability 8. Yes - all participants underwent the same semi structured interview and battery of psychopathology assessments 9. Can’t tell – a prevalence period was not specified 10. No – need a cut-off score for presence/absence of self-disorder which is not possible with EASE 11. Moderate internal validity and low external validity = moderate/high risk of bias | Moderate | Study limitations/risk of bias   - Some failure to develop and apply appropriate eligibility criteria   - Inclusion and exclusion criteria led to a high functioning, well-demarcated and pre-diagnosed sample, potentially introducing selection bias and affecting generalisability - >70 IQ, danish fluent, 9 school years, etc   - Was appropriate for study aims and interviewing process - Some flawed measurement of both exposure and outcome   - No outcome differences between group   - No assessment blinding – necessary to facilitate interview process   - Cross sectional study - No apparent failure to adequately control confounding   - Range of demographic features were reported and presented – only differences were in special needs school attendance (expected) - No apparent o inadequately short follow-up   - Cross sectional so no need for follow-up   Inconsistency of results   - Cannot comment on variance of point estimates - Overlap of confidence intervals only for age, gender, years of education, special needs school attendance and mental problems before age 16 – these recognised by author in general linear regression and deemed not significant predictors of EASE total score - All EASE scores statistically significant and only predictor (SPD diagnosis) of EASE total score showed p <0.05 and no CI overlap.   Indirectness of evidence   - Population is applicable to the systematic review   - Patients with SPD and ASD - The intervention is applicable to the systematic review   - EASE as self-disturbance assessment tool - The outcome measures are applicable to the systematic review   - Mean total self-disorder score and SD - No indirect comparison   - OMI   Imprecision   - No apparent imprecision   Publication bias  Magnitude of effect   - No RR to calculate   Dose-response curve   - No dose-response curve   Residual confounding |
| Nordgaard et al ^[33]^ | 2020 | Moderate | 1. Yes – Target population is patients with FRS so not representative of national population. In terms of SSD national population, FRS captured SZ but not specifically mentioned if range of different SSDs were captured. Some SPD participants were also captured. 2. No – Target population is the FRS patient population. Eligibility criteria meant no alcohol/substance abuse, history of brain injury, mental retardation, organic brain disorder, involuntarily admitted or forensic patient so could be less representative. Hospital patients so less generalisable. 3. Can’t tell/no – No mention of random selection of participants 4. Yes – likely minimal since dropouts were analysed and no differences in EASE was found so non-response bias likely minimal. No Comment on demographic characteristics 5. Yes – data was collected directly from participants via direct semi-structured interview 6. Yes – case definition was specified for FRS as Koehler’s definition of thought insertion, thought withdrawal, thought broadcast, thoughts aloud, delusions of control, delusional perception, commenting voice, & discussing voices 7. Yes – the EASE has proven validity and high interrater reliability 8. Yes – all participants underwent the same semi structured interview and battery of psychopathology assessments 9. Can’t tell – a prevalence period was not specified 10. No – need a cut-off score for presence/absence of self-disorder which is not possible with EASE 11. Moderate internal validity, moderate external validity = moderate risk of bias | Moderate | Study limitations/risk of bias   - Some failures to develop and apply appropriate eligibility criteria   - Eligibility criteria were specified and applied   - No healthy control groups   - Demographic characteristics table was provided with range of characteristics explored – groups appeared to match only on age, M/F ratio, married, and education – others and clinical features had statistically significant differences - Some minor flaws in measurement of both exposure and outcome   - Outcomes and exposure seem to be measured the same   - Interviewing clinicians were blinded   - Cohort - Possibly some failures to adequately control confounding   - Range of demographic features were reported and presented   - Unknown if all relevant confounders captured   - No adjustment for confounders in regression for ORs - No apparent inadequately short follow-up   - Follow-up of 5 years with is not apparently short   Inconsistency of results   - Can’t comment on variance for point estimates - CI: For whole sample, association of total EASE and odds of reporting FRS did not cross null value, and OR for 5-point difference in EASE did not cross null value. For no FRS sample, association of total EASE and odds of reporting FRS did cross null value, and OR for 5-point difference in EASE did cross null value. - P values: For whole sample, association of total EASE and odds of reporting FRS was p <0.05, and OR for 5-point difference in EASE was p <0.05 so both stat significant. For no FRS sample, association of total EASE and odds of reporting FRS was p >0.05, and OR for 5-point difference in EASE was p>0.05 so both not statistically significant. Statistically significant correlations between FRS, EASE total, neg symptoms, & perceptual disorders and all other clinical measures but intelligence not associated with any clinical measure   Indirectness of evidence   - Population is applicable to the systematic review   - Patients with FRS - The intervention is applicable to the systematic review   - EASE as self-disturbance assessment tool - The outcome measures are applicable to the systematic review   - EASE total score, negative symptoms, perceptual disorders (BSABS), intelligence, ORs for association of EASE (total and 5-point difference) and FRS - No indirect comparison   - No FRS   Imprecision   - No apparent imprecision   Publication bias  Magnitude of effect   - No RR to calculate   Dose-response curve   - No dose-response curve   Residual confounding |
| Nordgaard & Parnas ^[24]^ | 2014 | Moderate / High | 1. Yes – Target population is NAP and SPD only so appears representative of national population. In terms of SSD national population, NAP captures all SSDs but in this case some conditions like anxiety and major depression. That said also captures SPDs. 2. No – Target population is the SPD and NAP population. The sample is relatively representative of the target population, however the inclusion and exclusion criteria introduced selection bias by giving only a high functioning and well-demarcated sample. Hospital patients so less generalisable 3. Can’t tell/no - Participants were consecutively recruited but no mention of random selection. Favour for younger participants if too many participants further reducing randomness 4. Can/t tell – No follow up but some eligible participants refused to participate (16), later didn’t meet inclusion criteria (3), did not show (2), withdrew after (1). The diagnoses of those who did not participate were reported but no other demographic characteristics 5. Yes – data was collected directly from participants via direct semi-structured interview 6. Yes – case definition was specified as DSM-IV diagnostic criteria for each included condition 7. Yes – the EASE has proven validity and high interrater reliability 8. Yes – all participants underwent the same semi structured interview and battery of psychopathology assessments 9. Can’t tell – a prevalence period was not specified 10. No – need a cut-off score for presence/absence of self-disorder which is not possible with EASE 11. Moderate internal validity, moderate to low external validity = moderate/high risk of bias | Moderate | Study limitations/risk of bias   - Some failures to develop and apply appropriate eligibility criteria   - Eligibility criteria appropriately applied   - No healthy control groups   - Participants who were >65, organic brain disorder, IQ <70, substance misuse, aggression, severe psychosis, agitated, brain injury history or mental retardation were excluded.   - Other than the disorders they had, no demographic details were given on the participants that were not included (16) or dropped out   - EASE distribution was the same for age, gender, marital status and years of education - Possibly some flawed measurement of both exposure and outcome   - No outcome measurement differences between group   - No mention of interviewer blinding   - Cross sectional study/case series - No apparent failure to adequately control confounding   - Range of demographic features were reported and presented – only differences were in gender, (employment, educational level) – otherwise baseline characteristics fairly matched   - Unknown if all relevant confounders captured   - Multiple regression for confounding was performed on recorded variables – IQ didn’t affect but only IQ >70 included - No apparent inadequately short follow-up   - No follow up at this point – would be reported in subsequent study   Inconsistency of results   - Seemed to be some variance for point estimates of total and some domain EASE scores - CI not reported for many statistics - P values for EASE score differences were <0.05   Indirectness of evidence   - Population is applicable to the systematic review   - Patients with NAP and SPD - The intervention is applicable to the systematic review   - EASE as self-disturbance assessment tool - The outcome measures are applicable to the systematic review   - Mean total self-disorder score (EASE) - No indirect comparison   - OMI   Imprecision   - Nonapparent   Publication bias  Magnitude of effect   - No RR to calculate   Dose-response curve   - No dose-response curve   Residual confounding |
| Parnas et al ^[34]^ | 2003 | Moderate | 1. No – Target population is SZ and BD so not representative of national population. In terms of SSD national population, had SZ patients which form the largest proportion of patients with SSD but no mention of other SSDs and no people with SPDs 2. Cant tell/no – Target population is the SZ and BD population. The sample is relatively representative of the target population, but no eligibility criteria are given so cannot comment on how representative the resulting sample was. Hospital patients so less generalisable 3. Can’t tell/no – No mention of random selection of participants 4. Can/t tell – No follow up and no apparent dropouts so can’t comment on non-response bias. 5. Yes – data was collected directly from participants via direct semi-structured interview 6. Yes – case definition was specified as DSM-IV diagnostic criteria for SZ and BD 7. Yes – the BSABS has proven validity and high interrater reliability 8. Yes – all participants underwent the same semi structured interview and battery of psychopathology assessments 9. Can’t tell – a prevalence period was not specified 10. Yes – need a cut-off score for presence/absence of self-disorder which is possible with BSABS 11. Moderate to high internal validity, low external validity = moderate risk of bias | Low | Study limitations/risk of bias   - Failures to develop and apply appropriate eligibility criteria   - No eligibility criteria were specified   - No healthy control groups   - Demographic characteristics table provided but limited demographics   - Both groups varied on age only with statistical significance, but this did not correlate with scale scores   - Cannot otherwise comment on group matching but seems good - No apparent flawed measurement of both exposure and outcome   - No outcome measurement differences between group   - No mention of interviewer blinding but clinician blinding to the trial occurred   - Cross sectional study/case series - No apparent failure to adequately control confounding   - Some demographic features were reported and presented – only differences were in age – otherwise baseline characteristics fairly well matched   - Unknown if all relevant confounders captured   - Logistic regression performed and found no correlation with any demographic characteristic - No apparent inadequately short follow-up   - No follow up at this point   Inconsistency of results   - Seemed to be some variance for point estimates of total and some domain BSABS scores - CI: For univariate, CIs did not overlap for self-disorder and perceptual disorder ORs but did for other domains. For multivariate only self-disorder OR was significant and did not cross the null value for SZ status outcome - P values: For univariate, p values were <0.05 for self-disorder and perceptual disorder ORs but did for other domains. For multivariate, p values were <0.05 for self-disorder OR only   Indirectness of evidence   - Population is applicable to the systematic review   - Patients with SZ - The intervention is applicable to the systematic review   - BSABS as self-disturbance assessment tool - The outcome measures are applicable to the systematic review   - Mean total self-disorder score (BSABS), ORs - No indirect comparison   - BD   Imprecision   - Nonapparent   Publication bias  Magnitude of effect   - No RR to calculate   Dose-response curve   - No dose-response curve   Residual confounding |
| Parnas et al ^[5]^ | 2005 | Moderate | 1. Yes – Target population is SZ and SPD so not representative of national population. In terms of SSD population, captures all types of SSD and SPD so representative 2. Cant tell/no – Target population is the SZ and SPD population. The sample is relatively representative of the target population. Eligibility criteria meant no comparison condition, no organic brain disorder, no severe substance misuse and no psychiatric co-morbidity, potentially affecting how representative the resulting sample was. Hospital patients so less generalisable 3. Can’t tell/no – No mention of random selection of participants 4. Can/t tell – No follow up and no apparent dropouts so cannot comment on non-response bias. 5. Yes – data was collected directly from participants via direct semi-structured interview 6. Can’t tell/yes – case definition was specified as diagnostic criteria for SZ and SPD, but specific criteria used isn’t stated 7. Yes – the BSABS has proven validity and high interrater reliability 8. Yes – all participants underwent the same semi structured interview and battery of psychopathology assessments 9. Can’t tell – a prevalence period was not specified 10. Yes – need a cut-off score for presence/absence of self-disorder which is possible with BSABS 11. Moderate to high internal validity, moderate/low external validity = moderate risk of bias | Low | Study limitations/risk of bias   - Failures to develop and apply appropriate eligibility criteria   - Eligibility criteria were specified and applied   - No healthy control groups   - No demographic characteristics table provided and no discussion of matching and differences between groups - Some flawed measurement of both exposure and outcome   - Some missing data for different groups but attempts made to handle missing data   - No mention of interviewer blinding or clinician blinding   - Cross sectional study/case series - Failures to adequately control confounding   - No demographic features were reported and presented – only sex   - Unknown if all relevant confounders captured   - Binary logistic regression for different BSBABS domains between SZ and SPD - No apparent inadequately short follow-up   - No follow up at this point   Inconsistency of results   - Seemed to be some variance for point estimates of total and some domain BSABS scores - CI: For unadjusted, CIs did not overlap for all ORs except self-disorders, perceptual disorders and cenesthesia. For sex adjusted, only contact disorders, PANSS positive score, PANSS negative score, anxiety and affectivity ORs significant and did not cross the null value for SZ status outcome - P values: For unadjusted, p values were <0.05 for all ORs except self-disorders, perceptual disorders and cenesthesia. For sex adjusted, p values were <0.05 for contact disorders, PANSS positive and negative score, anxiety and affectivity ORs.   Indirectness of evidence   - Population is applicable to the systematic review   - Patients with SZ - The intervention is applicable to the systematic review   - BSABS as self-disturbance assessment tool - The outcome measures are applicable to the systematic review   - Mean total self-disorder score (BSABS), Odds ratios, PANSS, cognitive disorder, formal thought disorder - No indirect comparison   - SPD   Imprecision   - Possibly some imprecision   Publication bias  Magnitude of effect   - No RR to calculate   Dose-response curve   - No dose-response curve   Residual confounding |
| Parnas et al ^[35]^ | 2011 | Low | 1. Yes – Target population is SZ and SPD so not representative of national population. In terms of SSD population, both range of SZs and SPD so captures all types of SSDs and therefore representative 2. Yes – Target population is the SZ and SPD population. The sample is relatively representative of the target population. Eligibility criteria meant no comparison condition, no organic brain disorder, no severe substance misuse and no psychiatric co-morbidity, potentially affecting how representative the resulting sample was, but stabilised severe and aggressive participants were included. Hospital patients so less generalisable 3. Can’t tell – No mention of random selection of participants 4. Yes – Nonresponse bias minimal as dropout participant diagnoses reported and demographic characteristics compared and determined not significantly different 5. Yes – data was collected directly from participants via direct semi-structured interview 6. Yes – case definition was specified as ICD-10 diagnostic criteria for SZ and SPD and OMIs 7. Yes – the BSABS has proven validity and high interrater reliability 8. Yes – all participants underwent the same semistructured interview and battery of psychopathology assessments 9. Can’t tell – a prevalence period was not specified 10. Yes – need a cutoff score for presence/absence of self-disorder which is possible with BSABS 11. Moderate to high internal validity, moderate external validity = low risk of bias | Low | Study limitations/risk of bias   - Little to no failures to develop and apply appropriate eligibility criteria   - Eligibility criteria were specified and applied   - No healthy control groups   - Demographic characteristics table was provided, and groups were fairly well matched on all characteristics   - BD, melancholic depression and organic brain disorder were excluded but severely psychotic, aggressive participants were included once stabilised. - No apparent flawed measurement of both exposure and outcome   - Diagnostic conversion was recorded, and it was reported that demographic characteristics of those lost to follow up did not vary to those interviewed   - No mention of interviewer blinding or clinician blinding   - Cohort - No apparent failures to adequately control confounding   - A range of demographic features were reported and presented –   - Unknown if all relevant confounders captured   - Logistic regression on baseline variables at follow-up and did not vary - No apparent inadequately short follow-up   - Follow up seemed adequate at mean days 1889 days   Inconsistency of results   - Seemed to be minimal variance for point estimates of total and some domain BSABS scores - CI: Wide confidence intervals for OR of SSD conversion with high perplexity, self-disorder scores but also do not cross null value. Other scale score ORs crossed null so not significant - P values: P values <0.05 for OR of SSD conversion with high perplexity, self-disorder scores but also do not cross null value. Other scale score ORs p > 0.05 so not significant - OR calculated using Fischer’s exact. This is inappropriate for such a small sample size and explains the inflated OR calculated.   Indirectness of evidence   - Population is applicable to the systematic review   - Patients with SZ & SPD - The intervention is applicable to the systematic review   - BSABS as self-disturbance assessment tool - The outcome measures are applicable to the systematic review   - Mean total self-disorder score (BSABS), perplexity, perceptual disorders, PANSS, cognitive disorders, formal thought disorder, anxiety & affective symptoms, ORs - No indirect comparison   - OMI   Imprecision   - No apparent imprecision   Publication bias  Magnitude of effect   - No RR to calculate   Dose-response curve   - No dose-response curve   Residual confounding |
| Raballo & Maggini ^[36]^ | 2005 | Low | 1. Yes/no – Target population is SZ, MD and OCD so not representative of national population. In terms of SSD population, has SZ patients but no SPD and no mention of different types of SSD so fairly well represented but not the best 2. Yes – Target population is the SZ, MD and OCD population. No specific eligibility criteria so do not know how representative. But only stable patients in remission so could limit generalisability. Also, hospital patients only so limited. No organic brain disorder, no severe substance misuse potentially affecting how representative the resulting sample was, but stabilised severe and aggressive participants were included. Hospital patients so less generalisable 3. Can’t tell/no – No mention of random selection of participants 4. Can’t tell – No follow up so likely no none. Limited demographics 5. Yes – data was collected directly from participants via direct semi-structured interview 6. Yes – case definition was specified as DSM-IV diagnostic criteria for SZ and OCD and MD 7. Yes – the BSABS has proven validity and high interrater reliability 8. Yes – all participants underwent the same semi structured interview and battery of psychopathology assessments 9. Can’t tell – a prevalence period was not specified 10. Yes – need a cut-off score for presence/absence of self-disorder which is possible with BSABS 11. Moderate to high internal validity, moderate external validity = low risk of bias | Moderate | Study limitations/risk of bias   - Some failures to develop and apply appropriate eligibility criteria   - No specific eligibility criteria were specified but only DSM-IV diagnosed SZ, MD, OCD, stable remission, unchanged medication   - No healthy control groups   - Demographic characteristics table was provided, and groups were fairly well matched and not statistically different on all characteristics (except mean age) but demographics explored was limited - No apparent flawed measurement of both exposure and outcome   - Outcomes and exposure seem to be measured the same   - No mention of interviewer blinding or clinician blinding   - Cross sectional/case series - No apparent failures to adequately control confounding   - Some key demographic features were reported and presented   - Unknown if all relevant confounders captured   - Logistic regression not performed on baseline variables - No apparent inadequately short follow-up   - No follow up   Inconsistency of results   - Seemed to be some variance for point estimates of total and some domain BSABS scores - CI: Only the OR for cognitive disorders as a predictor of diagnostic attribution had CI which did not cross the null value. Had significant range - P values: Only the OR for cognitive disorders as a predictor of diagnostic attribution had p values <0.05 and so had statistical significance   Indirectness of evidence   - Population is applicable to the systematic review   - Patients with SZ - The intervention is applicable to the systematic review   - BSABS as self-disturbance assessment tool - The outcome measures are applicable to the systematic review   - Mean total self-disorder score (BSABS), ORs - No indirect comparison   - MD, OCD   Imprecision   - No apparent imprecision   Publication bias  Magnitude of effect   - No RR to calculate   Dose-response curve   - No dose-response curve   Residual confounding |
| Raballo et al ^[20]^ | 2011 | Moderate / Low | 1. Yes – Target population is SZ and SPD so not representative of national population. In terms of SSD national population, appears to be representative as SZ group has schizophrenia and other SSDs as well as SPD 2. Can’t tell/no – Target population is the SZ, SPD and OMIs population. No specific eligibility criteria so do not know how representative. Hospital patients so less generalisable. Also recruited vertically from 6 families, potentially making the sample less representative 3. Can’t tell/no – No mention of random selection of participants 4. Can’t tell – No follow up so likely no none. Limited demographics 5. Yes – data was collected directly from participants via direct semi-structured interview 6. Yes – case definition was specified as DSM-III diagnostic criteria for SZ, SPD, and OMIs 7. Yes – the BSABS has proven validity and high interrater reliability 8. Yes – all participants underwent the same semi structured interview and battery of psychopathology assessments 9. Can’t tell – a prevalence period was not specified 10. Yes – need a cut-off score for presence/absence of self-disorder which is possible with BSABS 11. Moderate to high internal validity, Moderate to low external validity = moderate/low risk of bias | Moderate | Study limitations/risk of bias   - Some failures to develop and apply appropriate eligibility criteria   - No specific eligibility criteria were specified   - Healthy control groups   - Demographic characteristics table was provided but limited characteristics explored   - Participants did not match on sex distribution and mean age (statistically significant difference) - No apparent flawed measurement of both exposure and outcome   - Outcomes and exposure seem to be measured the same   - Interviewing clinicians were blinded   - Cross sectional/case series - No apparent failures to adequately control confounding   - Some key demographic features were reported and presented   - Unknown if all relevant confounders captured   - Linear regression performed on baseline demographic variables – age (one variable not matched between groups) did show a significant, small negative association - No apparent inadequately short follow-up   - No follow up   Inconsistency of results   - Can’t comment on variance for point estimates - CI: SZ, SPD, OMI and age had ORs CIs for the absence/presence of self-disorders which did not cross the null for all variables except sex. Wide CI ranges for SZ - P values: SZ, SPD, OMI and age had ORs p values for the absence/presence of self-disorders which were >0.05 variables except sex. Shows statistical significance   Indirectness of evidence   - Population is applicable to the systematic review   - Patients with SZ. SPD, OMI - The intervention is applicable to the systematic review   - BSABS as self-disturbance assessment tool - The outcome measures are applicable to the systematic review   - BSABS domains in general linear model, ORs - No indirect comparison   - HC   Imprecision   - No apparent imprecision   Publication bias  Magnitude of effect   - No RR to calculate   Dose-response curve   - No dose-response curve   Residual confounding |
| Spark et al ^[37]^ | 2021 | Moderate | 1. Yes – Target population is FEP and UHR so not representative of the entire national population. In terms of SSD national population, FEP group had range of SSD types and UHR had borderline SSD conditions and SPD so representative 2. Yes/no - Target population is the FEP and UHR population. The study presents a table of demographic features of the sample. Participants were matched for all demographic features with the healthy controls. Participants with IQ <70, lack of proficiency with English and participants aged 15-25, with organic disorders, or previous psychiatric illness if UHR were excluded potentially affecting the representation of the sample. Outpatient so more generalisable 3. Can’t tell/no – No mention of random selection 4. Can’t tell – no apparent nonresponse bias as no dropouts 5. Yes – data was collected directly from participants via direct semi-structured interview 6. Yes – case definition was specified for UHR as attenuated psychotic symptoms, brief limited intermittent psychotic symptoms, trait vulnerability and deteriorating or chronic low functioning; and for FEP daily positive psychotic symptoms for longer than one week 7. Yes – the EASE has proven validity and high interrater reliability 8. Yes – all participants underwent the same semi structured interview and battery of psychopathology assessments 9. Can’t tell – a prevalence period was not specified 10. No – need a cut-off score for presence/absence of self-disorder which is not possible with EASE 11. Moderate internal validity, moderate external validity = moderate risk of bias | Moderate | Study limitations/risk of bias   - No apparent failures to develop and apply appropriate eligibility criteria   - Eligibility criteria appropriately applied   - Healthy control group but no clinical control group   - Healthy controls matched for age and sex – post hoc tests showed SZ group younger than HCs   - Participants who were older than 15-25, IQ <70 and not fluent in English, had organic brain disorders, past psychiatric disorder (UHR) were excluded – may introduce bias - Possibly some flawed measurement of both exposure and outcome   - No outcome differences between group   - No mention of interviewer blinding   - Cross sectional study - No apparent failure to adequately control confounding   - Some demographic features were reported and presented – only differences were in age for SZ group – otherwise baseline characteristics matched   - Unknown if all relevant confounders captured   - No apparent regression was performed to adjust for confounding - No apparent inadequately short follow-up   - No follow up   Inconsistency of results   - Some variances in point estimates for mean EASE and other recorded outcome measure scores - CI not reported for many statistics - All EASE scores statistically significant, except demarcation/transivitism score   Indirectness of evidence   - Population is applicable to the systematic review   - Patients with SZ within FEP and UHR groups - The intervention is applicable to the systematic review   - EASE as self-disturbance assessment tool - The outcome measures are applicable to the systematic review   - Mean total self-disorder score (EASE) - No indirect comparison   - HC   Imprecision   - No apparent imprecision   Publication bias  Magnitude of effect   - No RR to calculate   Dose-response curve   - No dose-response curve   Residual confounding |
| Svendsen et al ^[38]^ | 2020 | Moderate / Low | 1. Can’t tell/yes – Target population is SZ, BD and OMI only so not representative of national population. In terms of SSD population, a loose definition was adopted so a range of SSDs were captured. No SPD. This taken from initial study as this is a follow up and so not specifically mentioned in this study. 2. Yes – Target population is the SZ population. SZ, BD and OMI group well represented in sample. Group sizes not matched. The study presents a table of demographic features of the sample. There were no stat significant differences for age or affective symptoms. Efforts to include patients with substance misuse disorder if not currently using. Cannot tell if inpatient or outpatient. Near epidemiological sample with some attrition but not apparently due to bias 3. No – there is no mention of random selection. Interviewers were also not blinded but Bonferroni post-hoc tests performed to minimise impact. Analysing statistics were 4. Yes – in follow up study there was no significant differences between responders and non-responders 5. Yes – data was collected directly from participants in semi-structured interviews 6. Yes – case definition as the DSM-IV diagnosis of SZ, BD or OMI and confirmed by research SCID 7. Yes – the EASE has proven validity and high interrater reliability 8. Yes - all participants underwent the same semi structured interview and battery of psychopathology assessments 9. Can’t tell – a prevalence period was not specified 10. No – need a cut-off score for presence/absence of self-disorder which is not possible with EASE 11. Moderate internal validity and moderate to high external validity = moderate to low risk of bias | Moderate | Study limitations/risk of bias   - Some minor failures to develop and apply appropriate eligibility criteria   - Eligibility criteria appropriately applied but participants which did not fully meet original criteria were added to increase statistical power. Were still from the same setting   - No healthy control groups   - Managed to include patients with previous substance disorders by allowing patients with history of substance abuse but no recent use   - Exclusion of participants with IQ <70 it specified – follow up to Haug 2012 and Svendsen 2018 which specify these criteria which could be less generalisable - Some minor flaws in measurement of both exposure and outcome   - No outcome differences between group   - No assessment blinding – necessary to facilitate interview process – Bonferroni’s post-hoc tests to minimise impact of this on EASE scores   - Cohort but only follow up – more cross sectional   - No differences in demographics and clinical characteristics of those that did and did not attend follow up - No apparent failure to adequately control confounding   - Some demographic features were reported and presented – age, sex and in other studies were fairly well matched - No apparent inadequately short follow-up   - Follow up to cohort   Inconsistency of results   - Cannot comment on variance of point estimates - Confidence intervals not reported for most statistics. - Between SS and NSS groups: EASE score, GAF symptom score, GAF function score, SPS score, PANSS positive score, PANSS negative score, PANSS DIS had p values <0.05. PANSS DEP, PANSS EXC had p values >0.05   Indirectness of evidence   - Population is applicable to the systematic review   - Patients with SZ (SS group) - The intervention is applicable to the systematic review   - EASE as self-disturbance assessment tool, Assessment of Sense of Agency - The outcome measures are applicable to the systematic review   - Mean total self-disorder score and SD, Mean SOC score, EASE score SOC score association - No indirect comparison   - Patients with BD or OMI (non-SS group)   Imprecision   - No apparent imprecision   Publication bias      Magnitude of effect   - No RR to calculate   Dose-response curve   - No dose-response curve   Residual confounding |

Table detailing the rationale for risk of bias and quality of evidence rating for included studies. Risk of bias was determined with a risk of bias assessment tool for prevalence and quality of evidence was determined by GRADE (grading of recommendations, assessments, development, and evaluation) handbook.

Abbreviations: ASD = autism spectrum disorder, ASE = anomalous self-experience, BD = bipolar disorder, BPRS = brief psychiatric rating scale, BSABS = Bonn scale for the assessment of basic symptoms, CAARMS = comprehensive assessment of at risk mental states, EASE = Examination of anomalous self-experiences, EAWE = examination of anomalous world-experiences, DSM = diagnostic and statistical manual, F = female, FEP = first episode psychosis, FRS = first rank symptoms, GAF = global assessment of functioning, HC = healthy control, ICD = international classification of diseases, M = male, MD = mood disorder, NAP = non-affective psychosis, NSS = non-schizophrenia spectrum, OCD = obsessive compulsive disorder OMI = other mental illness, OPCRIT = operational criteria checklist, PANSS = positive and negative syndrome scale, RR = , SANS = scale for the assessment of negative symptoms, SD = self-disorder, SOC = sense of coherence, SOFAS = social and occupational functioning assessment scale, SPD = schizotypal personality disorder, SS = schizophrenia spectrum, SSD = schizophrenia spectrum disorder, SZ = schizophrenia, UHR = ultra-high risk for psychosis, YMRS = young mania rating scale.

**Supplementary Fig. 1** Sensitivity analysis of the likelihood of developing self-disorders in schizophrenia spectrum and control groups, as measured with the Bonn scale for the assessment of basic symptoms (BSABS).

**

Studies are grouped by outcome measure. **a)** describes the odds ratios of developing self-disorders in schizophrenia spectrum and control groups when the Parnas et al. 2003 study was removed. **b)** describes the odds ratios of developing self-disorders in schizophrenia spectrum and control groups when the Parnas et al. 2011 study was removed. **c)** describes the odds ratios of developing self-disorders in schizophrenia spectrum and control groups when the schizotypal disorder subgroup of the Raballo et al. 2011 study was removed. **d)** describes the odds ratios of developing self-disorders in schizophrenia spectrum and control groups when the schizophrenia subgroup of the Raballo et al. 2011 study was removed. **e)** describes the odds ratios of developing self-disorders in schizophrenia spectrum and control groups when all potential outliers were removed. Data shown as log odds ratio ± SE are representative of two independent samples; values are significant if p<0.05.

Abbreviations: BSABS = Bonn scale for the assessment of basic symptoms, EASE = Examination of anomalous self-experiences, HC = healthy control, MD = mood disorder, NAP = non-affective psychosis, OCD = obsessive compulsive disorder, OMI = other mental illness, SD = self-disorder, SPD = schizotypal personality disorder, SZ = schizophrenia.

**Supplementary Material 1** Detailed description of the results from the sensitivity analysis (*Supplementary Figure 1)*.

Panels *a)* to *d)* of *Supplementary Figure 1* describe the odds ratio effect sizes when each potential outlier is removed. As panel *a)* shows, removal of Parnas et al 2003 ^[34]^ reduced the pooled effect size to Hedge’s *g* = 5.369 (95% CI 2.211-13.037) and increased heterogeneity (*I^2^* = 71%). In panel *b)*, the removal of Parnas et al 2011 ^[35]^ caused the pooled effect size to decrease to Hedge’s *g* = 4.902 (95% CI 2.076-11.573). Surprisingly, heterogeneity increased to *I^2^* = 70%. Panel *c)* shows that the pooled effect size reduced upon removal of the schizophrenia subgroup in the Raballo et al 2011 study, ^[20]^ adjusting to Hedge’s *g* = 4.284 (95% CI 1.964-9.344). Heterogeneity was reduced but remained high (*I^2^* = 59%). In panel *d)*, removing the schizotypal personality disorder subgroup in the Raballo et al 2011 study ^[20]^ caused the pooled effect size to decrease to Hedge’s *g* = 4.603 (95% CI 1.855-11.417). Heterogeneity was almost unchanged (*I^2^* = 65%).

Panel *e)* of *Supplementary Figure 1* displays the effect size when all potential outliers are removed from the model. With all potential outliers removed, the pooled effect size reduced by over half (Hedge’s *g* = 2.269, 95% CI 1.143-4.504). Heterogeneity was low (*I^2^* = 0%) but this highlights an issue with this effect size. With all potential outliers removed only two studies were included within the model, meaning the sample size was small and the power of the effect size was lower.

**Supplementary Material 2** Detailed description of the methodology for data collection.

An example search strategy can be found in *Supplementary Material 3*. This search strategy contained the key words “self-disorder”, “self-disturbance” and “anomalous self-experience” paired with OR as these terms are used interchangeably in the literature. The key word “schiz*” was used to ensure that all variations of SSD were captured within the search. Combining “assessment” and “screening” with OR ensured that all papers using the assessment tools were gathered, even if the purpose for using them differed between individual studies. The grey literature databases lacked an advanced search function and so variations of key words were used to search for studies. Zotaro was used as a reference managing software for this systematic review.

Before beginning screening, duplicate studies identified in the electronic search were removed. In the screening phase, all identified studies had their titles and abstracts screened independently by the researcher (S.B.) and research supervisor (C.H.). In the review phase, the remaining full text articles were read and evaluated for inclusion against the eligibility criteria by the researcher (S.B.) and research supervisor (C.H.). The eligibility criteria for inclusion and exclusion are summarised in *Supplementary Table 1*. Any discrepancies in selected studies, during either the screening or full article review phase, were resolved through meetings with an independent third-party researcher (R.R.). This was done to minimise selection and publication bias considering the small research team. Each stage of data collection was recorded within the PRISMA Flowchart (*Figure 1*). ^[47]^

**Supplementary Material 3** Search strategy for Medline, Embase, & PsychINFO.

(Schiz*)

AND self-disorder) OR self-disturbance) OR anomalous self-experience)

AND assessment) OR clinical assessment) OR screening)

**Supplementary Material 4** Detailed description of the methodology for quality of evidence and risk of bias assessments.

The quality of evidence rating for each included study was graded as either “high”, “moderate”, “low”, or “very low” depending on their accordance with high-quality observational study criteria. The Grading of Recommendations, Assessment, Development, and Evaluation (GRADE) recommends a base rating of “low” for all non-randomised trials. ^[48]^ However, the authors of this review thought that given the purpose of this review, the relative scarcity of evidence, and that most included studies employ a new type of aetiology study, all included studies would instead be assessed with a base rating of “moderate”.

The 10-item risk of bias assessment tool facilitated the assessment of selection, nonresponse, measurement, and analytic bias in included studies. ^[49]^ A subjective summary assessment rating of either “low”, “moderate”, or “high” was then generated for each study.

**Supplementary Material 5** GRADE handbook and grading quality of evidence.

<https://gdt.gradepro.org/app/handbook/handbook.html>

**Quality of evidence grades**


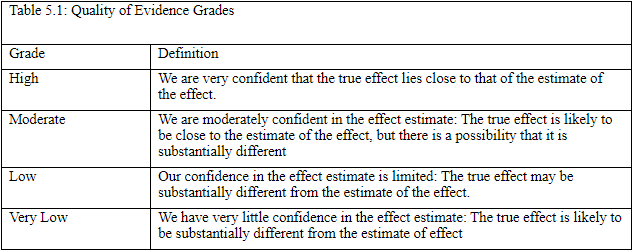


**Quality of evidence assessment based of study design**


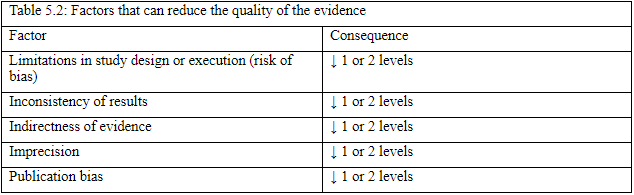

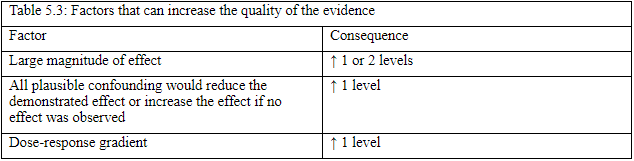


**Supplementary Material 6** Risk-of-bias assessment tool for prevalence studies.

<https://pubmed.ncbi.nlm.nih.gov/22742910/>


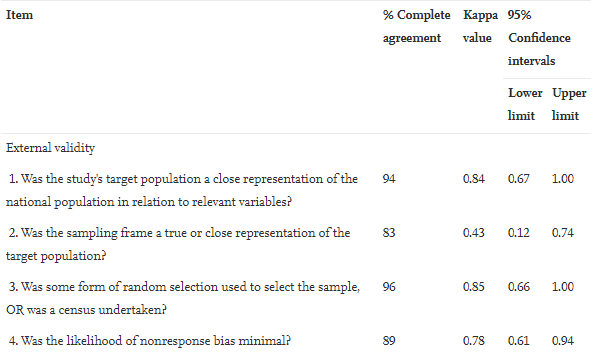

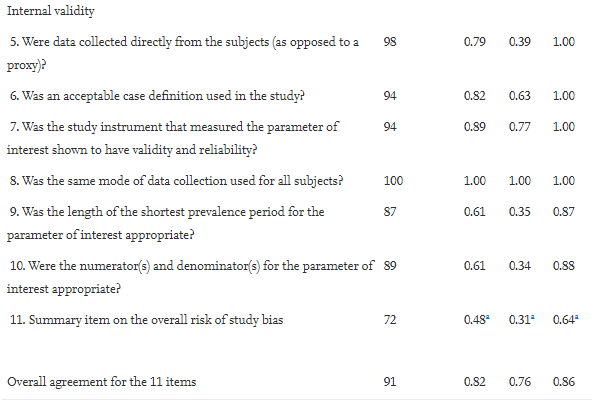


*
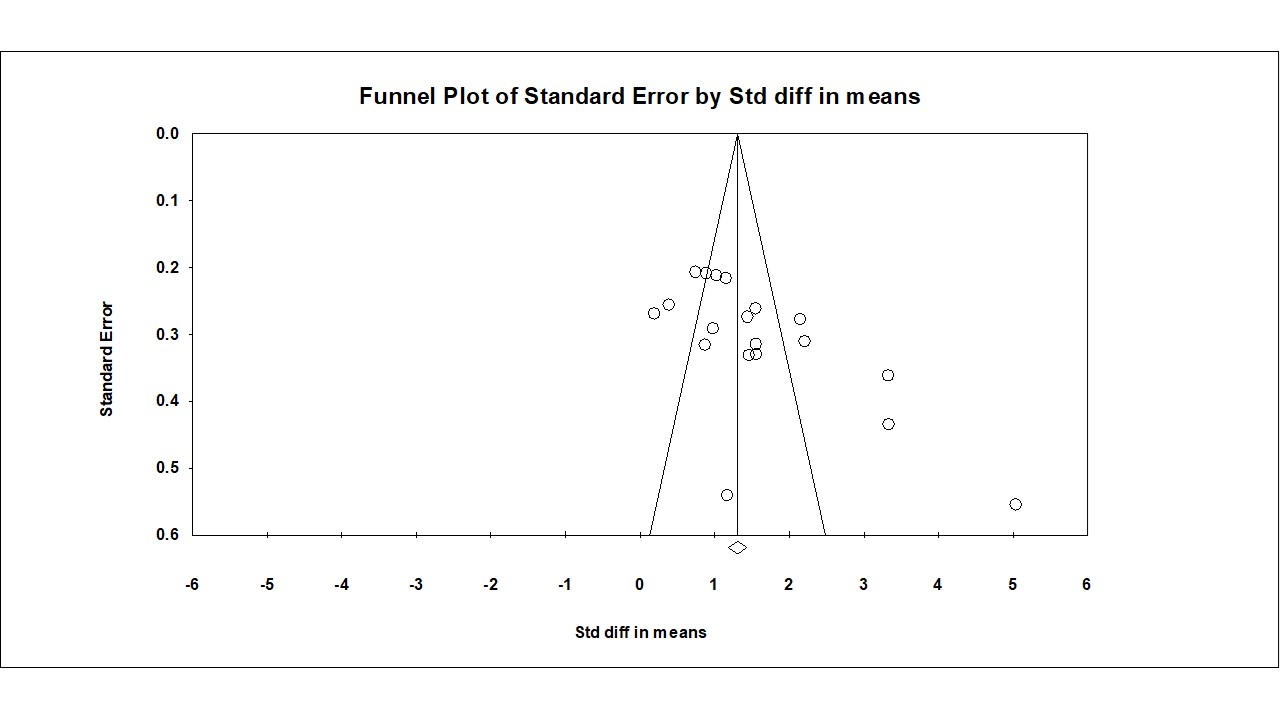
*

**Supplementary Material 7** Funnel plot for all SD dichotomous scores.
